# Dioecy and chromosomal sex determination are maintained through allopolyploid speciation in the plant genus *Mercurialis*

**DOI:** 10.1371/journal.pgen.1010226

**Published:** 2022-07-06

**Authors:** Melissa A. Toups, Beatriz Vicoso, John R. Pannell

**Affiliations:** 1 Department of Ecology and Evolution, University of Lausanne, Lausanne, Switzerland; 2 Institute of Science and Technology Austria, Klosterneuburg, Austria; Stockholm University, SWEDEN

## Abstract

Polyploidization may precipitate dramatic changes to the genome, including chromosome rearrangements, gene loss, and changes in gene expression. In dioecious plants, the sex-determining mechanism may also be disrupted by polyploidization, with the potential evolution of hermaphroditism. However, while dioecy appears to have persisted through a ploidy transition in some species, it is unknown whether the newly formed polyploid maintained its sex-determining system uninterrupted, or whether dioecy re-evolved after a period of hermaphroditism. Here, we develop a bioinformatic pipeline using RNA-sequencing data from natural populations to demonstrate that the allopolyploid plant *Mercurialis canariensis* directly inherited its sex-determining region from one of its diploid progenitor species, *M*. *annua*, and likely remained dioecious through the transition. The sex-determining region of *M*. *canariensis* is smaller than that of its diploid progenitor, suggesting that the non-recombining region of *M*. *annua* expanded subsequent to the polyploid origin of *M*. *canariensis*. Homeologous pairs show partial sexual subfunctionalization. We discuss the possibility that gene duplicates created by polyploidization might contribute to resolving sexual antagonism.

## Author summary

Polyploidization, or genome duplication, is widespread among plants, with consequences that include gene loss, genome reorganization, and large-scale changes in gene expression. These changes could also affect species with separate sexes, with implications for sex determination and the evolution of sexual dimorphism. In some plants, polyploidy may disrupt the mechanism of sex determination, potentially bringing about a transition to hermaphroditism. In others, such as the wind-pollinated annual plant *Mercurialis canariensis*, dioecy has been maintained through allopolyploidization (i.e. genome duplication associated with hybridization). Here, we show that *M*. *canariensi*s arose via hybridization between the dioecious relative *M*. *annua* and an unidentified, possibly extinct, second species. We also demonstrate that *M*. *canariensis* inherited its sex-determining region from *M*. *annua*. Finally, a small number of gene duplicates created by polyploidization have diverged in gene expression between males and females of *M*. *canariensis*. Our finding suggests that expression of each of the duplicated loci may have been differentially optimized for each sex. This possibility of ‘sexual subfunctionalization’ further highlights the dynamic interplay between polyploidization, sex-determination, and the evolution of sexual dimorphism.

## Introduction

It is estimated that ~30% of plant species also have recent polyploid ancestry [1,2]. These increases in ploidy can have wide-ranging consequences, including impacts on biogeography and ecology [3,4], rates of diversification [5–7], and genome evolution [8,9]. Whereas the importance and success of polyploidy in plants cannot be disputed, new polyploid lineages that persist eventually become re-diploidized, initially in terms of patterns of segregation [10,11], but ultimately also in terms of genome size [12,13], gene content, and gene expression [14]. For most genes, diploidization of polyploid genomes involves the loss of one of their duplicated copies through a process of ‘fractionation’ [14–16], which may be strongly influenced by differences in expression levels between homeologs [17,18]. Such fractionation is typically not random, so that one ‘dominant’ subgenome retains more genes than the other [19,20]. While gene loss appears to be the most likely fate of one or other of redundant copies in polyploid lineages, some genes may be retained via subfunctionalization [21], where each copy evolves a different pattern of expression (e.g., at different times during cellular or organ development, or in different cell or organ types; [22,23]).

The possibility of subfunctionalization is particularly interesting in dioecious plants, because it may contribute to the resolution of sexual antagonism [24,25], with one homeolog expressed more in males and the other more in females. This process of ‘sexual subfunctionalization’ has been detected in gene duplicates of diploid species across a broad phylogenetic scale, including Drosophila [26], brown algae [27], and mice [28], but it would seem to be particularly plausible in dioecious polyploid species, with each of the two homeologs expressed at a level closer to the phenotypic optimum of one sex or the other. Given that dioecious plants frequently display sexual dimorphism for a wide range of physiological, morphological, defense and life-history traits (summarized in [29]), the compartmentalization of the genome into two subgenomes in dioecious polyploids could overcome genetic correlations between the sexes that might otherwise limit the optimization of distinct male and female phenotypes. Because sexual subfunctionalization implies the retention of both gene copies after polyploidization, we might also expect the evolution of subgenome dominance to be less important in dioecious species.

Genome duplication in dioecious species also has implications for the evolution of sexual systems and sex determination. First, polyploidy can cause a minority cytotype disadvantage, where the lack of mates selects for self-fertile hermaphrodites [30,31]. And second, the substantial reorganization of the genome through polyploidization (including aneuploidies and chromosome rearrangements) can lead to a disruption of meiosis [30,32,33], which may cause the breakdown of the existing genetic sex-determination mechanisms to provoke a transition from dioecy to hermaphroditism [30,34], notably in species in which genome duplication interferes with sex determined by an X:A (or Z:A) balance, with the potential generation of ‘intersexes’ and hermaphroditism [32,34]. For instance, in species of both *Silene* and *Rumex* that have XY sex chromosomes, the progeny of synthetic polyploids are hermaphroditic [34]. Other species seem to maintain dioecy after polyploidization (e.g., *Silene*, *Rumex*, *Bouteloua*, *and Atrichum*; [30,35–38], but little is known about how this occurs. Outside of angiosperms, the maintenance of a sex-determining region through polyploidization has been suggested for sturgeon [39] and mosses [40]. While these studies illustrate the potential effect of polyploidy on the breakdown of dioecy, polyploidization can also precipitate the evolution of dioecy from hermaphroditism [30,41].

Only a few diploid/polyploid pairs have been thoroughly examined for their sex determination. The most well-studied taxa are wild strawberries of the genus *Fragaria* [42–49]. Dioecious species in *Fragaria* have octoploid genomes that consist of four distinct subgenomes, with little recombination between homeologous chromosomes [50]. The sex-determining region consists of a female-specific ‘cassette’ on the W chromosome that contains a candidate sex-determining gene [42]. The cassette is hypothesized to have evolved once, and then to have jumped between homeologous chromosomes in different species [42]. However, although sex determination in *octoploid* populations of *Fragaria* is well-characterized, it is unclear when separate sexes first arose. In persimmon, sex is determined in diploids by the autosomal gene MeGI and its Y-linked paralog OGI [51,52], but expression of OGI is disrupted in polyploids that may be responsible for reversion to hermaphroditism–although some polyploid lineages have secondarily re-established dioecy through the reactivation of OGI [53]. In contrast to these examples, the polyploid descendants of other diploid dioecious species appear to have maintained dioecy. In cases that involve allopolyploid hybridization between two diploid dioecious parents, it is pertinent to ask which of the two precursors contributed the sex determination system, and how the sex chromosomes might have evolved during the subsequent process of diploidization. The mainly European and north African clade of annual species of the genus *Mercurialis* presents one such case.

The genus *Mercurialis* shows remarkable variation in both its sexual system and ploidy [31]. Dioecy is ancestral in the genus, present in perennial species, but monoecy has evolved in polyploid annuals [31,54,55]. Polyploidization of diploid *M*. *annua* produced a monoecious tetraploid population, which subsequently hybridized with the *M*. *huetii*, the diploid sister species of *M*. *annua*, yielding hexaploid populations (still referred to as *M*. *annua*, or *M*. *ambigua*) with an androdioecious sexual system (the maintenance of males with hermaphrodites) [55]. Crosses among species [56] suggest that sex in hexaploid *M*. *annua* is determined by the same XY sex-chromosomal system as that characterized for diploid *M*. *annua*. Obbard et al [57] described a new dioecious species, *M*. *canariensis* (confined to the Canary Islands), which has a life history and morphology very similar to that of *M*. *annua*. However, while dioecious *M*. *annua* is diploid, *M*. *canariensis* is tetraploid [57]. The fact that *M*. *canariensis* is polyploid and dioecious is noteworthy because all other polyploid annuals in *Mercurialis* are monoecious. Obbard et al. [57] inferred that *M*. *canariensis* was an allotetraploid hybrid between diploid *M*. *annua* and a likely extinct progenitor that may also have been dioecious. It is not known which of the two putative progenitors of *M*. *canariensis* contributed the sex-determining locus.

**Fig 1:**
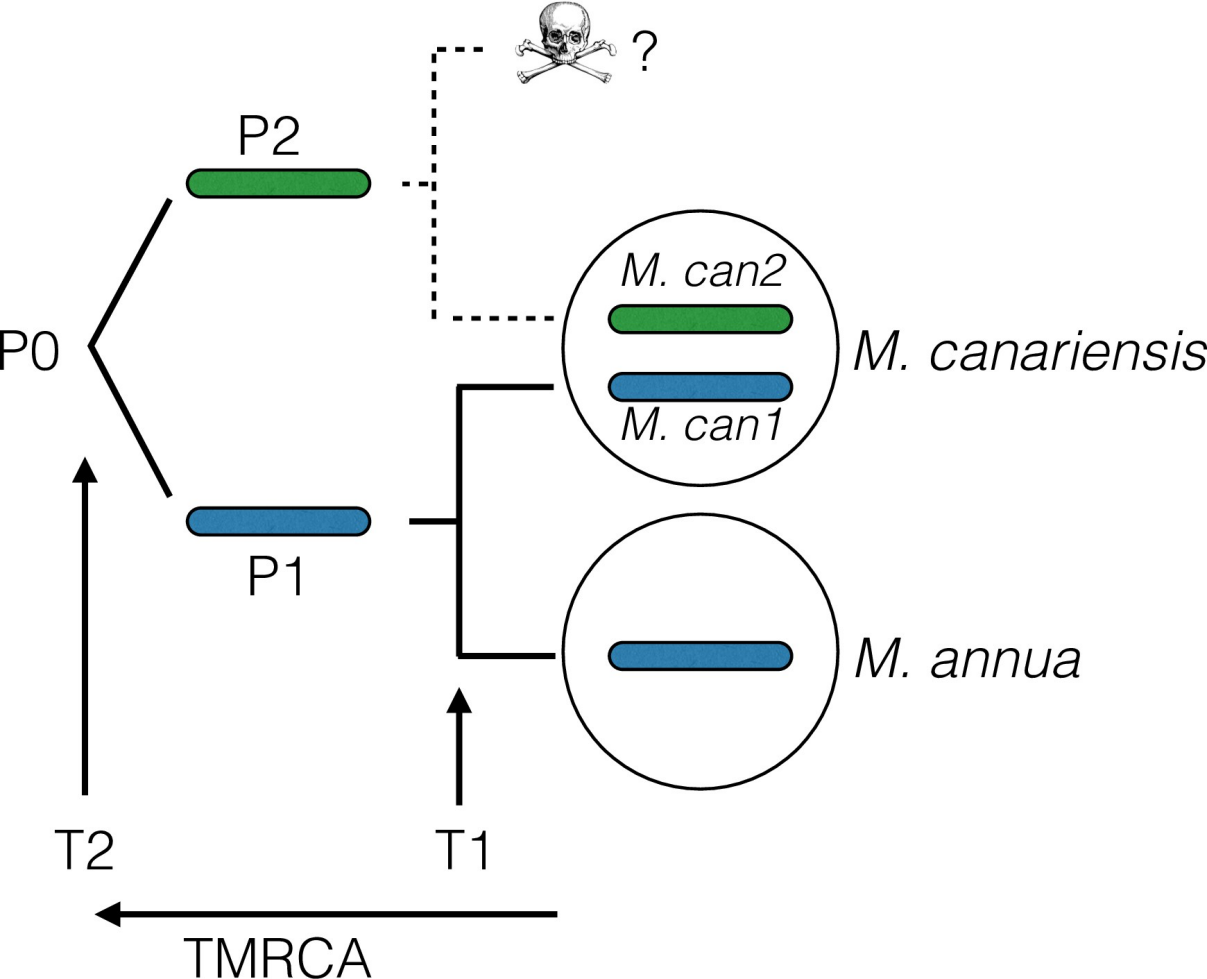
Conceptual approach for identifying the putative progenitors of each of the two subgenomes of *M*. *canariensis*, *M*. *can1* and *M*. *can2*. P0 is the most recent common ancestor (MRCA) of the two progenitors of allopolyploid *M*. *canariensis*, P1 is the progenitor of the first subgenome of *M*. *canariensis*, *M*. *can1*, and the MRCA of *M*. *can1* and extant diploid *M*. *annua*. P2 is the progenitor of the second subgenome of *M*. *canariensis*, *M*. *can2*, and a second likely extinct lineage. T1 and T2 are the times to the most recent common ancestors (TMRCA), indicated by the coalescence time arrow. We assigned transcripts from *M*. *canariensis* to one or other of the two subgenomes on the basis of silent-site divergence, dS, from the homologous sequence in *M*. *annua*: we inferred that transcripts with lower dS were derived from P1, whereas those with a higher dS were derived from P2 (because they share the more ancient MRCA with *M*. *annua*, P0).

Here, we use long-read RNA sequencing and expression data to re-examine the evolutionary origins of allotetraploid *M*. *canariensis* and to identify which of its two diploid progenitors contributed the sex chromosomes. We based our analysis on full-length transcripts from polyploid *M*. *canariensis*, thus overcoming the problem of assembling chimeric transcripts derived from different homeologs. As set out in Fig 1, our approach involved attributing the full-length transcripts to one of the two subgenomes of *M*. *canariensis*, *M*. *can1* and *M*. *can2*, on the basis of differences in inferred times to their common ancestor, either with each other or with an outgroup, the putative progenitor *M*. *annua*. Our analysis confirmed that diploid *M*. *annua* was one of the two progenitors of *M*. *canariensis*, as inferred by Obbard et al. [55]. It also identified the *M*. *can1* subgenome as the more closely related to present-day *M*. *annua* (P1 in Fig 1), and the *M*. *can2* subgenome as derived from the unknown progenitor (P2). With the robust identification of the two subgenomes of *M*. *canariensis*, we then sought evidence for biased gene expression and gene retention in *M*. *canariensis*, including the possible evolution of subgenome dominance. We also asked whether such patterns are consistent with a model of sexual subfunctionalization.

## Results

### Transcriptome assembly and homeolog identification

To obtain full-length transcripts of *Mercurialis canariensis*, we used PacBio long-read technology to sequence the RNA from the mature leaves of a female plant grown in the University of Lausanne glasshouses. The Isoseq pipeline identified 24,374 full-length transcripts (all datasets available in [58]). As this approach identifies and retains multiple isoforms per gene, we developed a pipeline to select only the longest isoform (See S1 Fig; all pipelines available in [59]). We used an all-vs-all blat to identify transcripts with high sequence identity to form transcript clusters, and we selected the longest isoform per cluster; this resulted in the identification of 12,918 transcripts for *M*. *canariensis*. We applied the same pipeline to select the longest isoform in the published *M*. *annua* transcriptome [60], from which we retained 21,230 of the 39,302 transcripts originally identified.

Next, we extracted homeologous sequence pairs derived from each of the two subgenomes from the reduced transcriptome of *M*. *canariensis* (i.e., consisting of only the longest isoform per gene cluster). To identify homeologs, we again used an all-vs-all blat within the reduced *M*. *canariensis* transcriptome and assigned reciprocal best blat hits to homeolog pairs. The median silent-site divergence, dS, between the putative homeologs was 0.14, consistent with a recent polyploidization event between two recently diverged parental lineages [61]. Each *M*. *canariensis* transcript was then mapped to the reduced *M*. *annua* transcriptome using blat, and dS was calculated between the *M*. *canariensis* transcript and the *M*. *annua* best hit (S1 Fig). We retained reciprocal best hit pairs as homeologs from our all-vs-all blat within *M*. *canariensis* if they shared a best hit in the *M*. *canariensis*-*M*. *annua* blat. This resulted in 3,135 putative homeolog pairs.

### The *M*. *annua* lineage is one of the ancestors of *M*. *canariensis*

**Fig 2:**
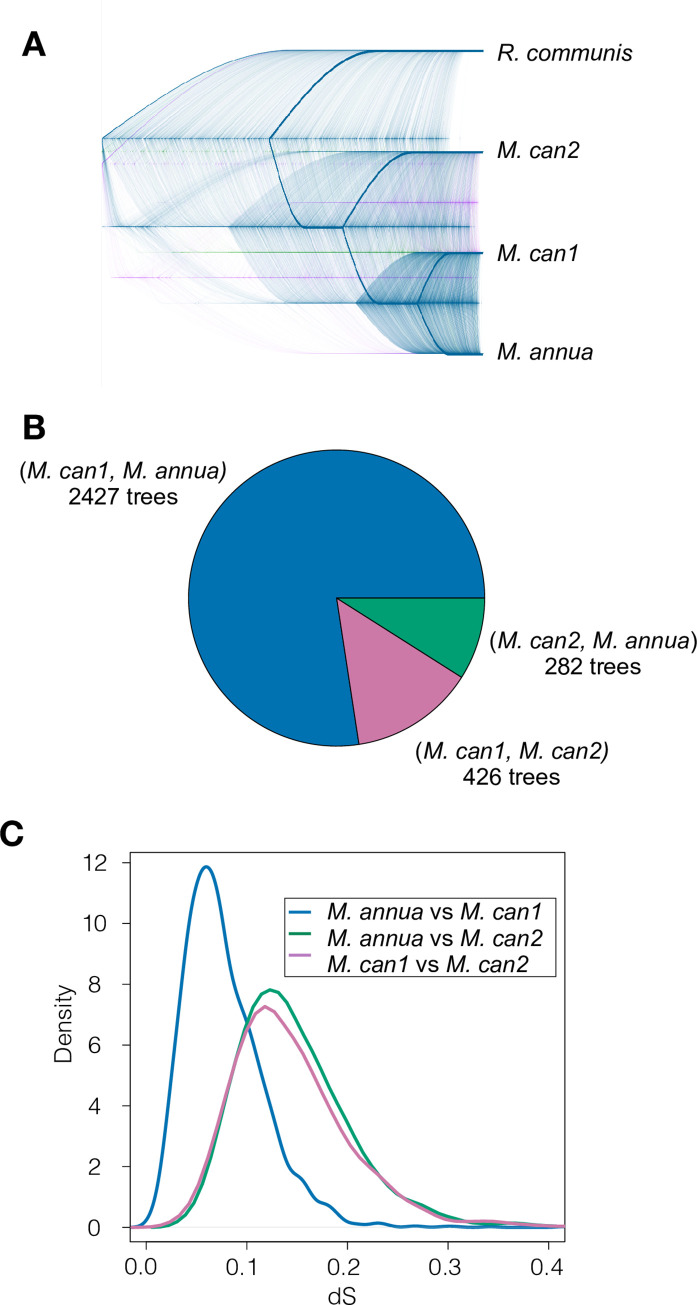
The *M*. *can1* subgenome is sister to *M*. *annua*. (A) Gene trees constructed from 3,135 homeolog pairs in *M*. *canariensis* and their orthologs in *M*. *annua* and *R*. *communis*. Blue, pink and green gene trees indicate those in which *M*. *can1* is sister to *M*. *annua*, *M*. *can1* is sister to *M*. *can2*, and *M*. *can2* is sister to *M*. *annua*, respectively. (B) Pie chart showing the proportion of trees in each category, and their respective topologies. (C). Density distribution of dS for *M*. *annua* vs. *M*. *can1* subgenome (blue), *M*. *annua* vs. *M*. *can2* subgenome (green), and *M*. *can1* vs. *M*. *can2* subgenomes (pink) among the 2,427 trees with the hypothesized topology.

We used dS from orthologous sequences in *M*. *annua* to assign each homeolog to either subgenome *M*. *can1* or *M*. *can2* (Fig 1). We preliminarily assigned the homeolog with the lower dS to the *M*. *can1* subgenome (Fig 1; derived from *M*. *annua*), and the homeolog with the higher dS to the *M*. *can2* subgenome (derived from an unsampled and likely extinct progenitor; Fig 1). We constructed gene trees to test the hypothesis that *M*. *annua* was the progenitor of the *M*. *can1* subgenome. For each homeolog pair, we included putative *M*. *can1* and *M*. *can2* homeologs, their ortholog in *M*. *annua*, and the corresponding 1:1 ortholog between *M*. *annua* and the more distant outgroup species *Ricinus communis*. The majority of trees (2427/3135 = 77%) had the expected topology of (((*M*. *can1*, *M*. *annua*), *M*. *can2*), *R*. *communis*) (Fig 2A and 2B).

We then examined the dS distributions of only those homeologs whose gene trees resulted in the expected topology. (Discordant topologies might be the result of assembly errors, or gene movement between subgenomes, e.g., due to recombination between homeologs.) First, we confirmed that the mean dS between *M*. *annua* and *M*. *can2* was significantly larger than between *M*. *annua* and *M*. *can1* (Fig 2C; Wilcoxon test, p < 2.2 x 10^−16^). Furthermore, if the *M*. *can1* subgenome was derived from *M*. *annua*, and our subgenome assignments are correct, then the divergence between *M*. *can1* and *M*. *can2* should be approximately equal to the divergence between *M*. *annua* and *M*. *can2* (Fig 1). Consistent with this hypothesis, we detected no difference between these two distributions (Fig 2C; Wilcoxon test, p = 0.067). Only homeologous pairs with the tree structure shown in Fig 2A were included in subsequent analyses.

To test the robustness of our results, we also employed an alternative method of homeolog subgenome assignment. Pairs were defined as above (having a shared a best hit in the *M*. *canariensis*-*M*. *annua* blat), but were filtered only on the basis of our hypothesized gene tree. The member of the pair that was inferred as sister to *M*. *annua* was assigned to the *M*. *can1* subgenome, while other was assigned to the *M*. *can2* subgenome. This more inclusive method resulted in 2709 pairs. Importantly, all of our results were consistent between the two methods (S2 and S3 Figs).

### *M*. *canariensis* inherited the SDR from *M*. *annua*

To determine the location of the sex-determining region in *M*. *canariensis*, we used paired-end short-read RNA-sequencing from six males and six females sampled from six populations in Tenerife. Previous work by Veltsos et al. [60] used a combination of linkage mapping and data from natural populations to demonstrate that the sex-determining region of *M*. *annua* is on linkage group 1. We thus obtained linkage group assignments for *M*. *annua* transcripts from Veltsos et al. [60]. Of the 21,230 transcripts in the reduced transcriptome of *M*. *annua*, 8,181 could be assigned to linkage groups. We then used the *M*. *annua* ortholog position in the corresponding linkage group to assign *M*. *canariensis* homeologs to a genomic location in either the *M*. *can1* or *M*. *can2* subgenomes, as previously inferred. This resulted in 1,471 homeologous pairs assigned to linkage group positions in *M*. *canariensis*.

**Fig 3:**
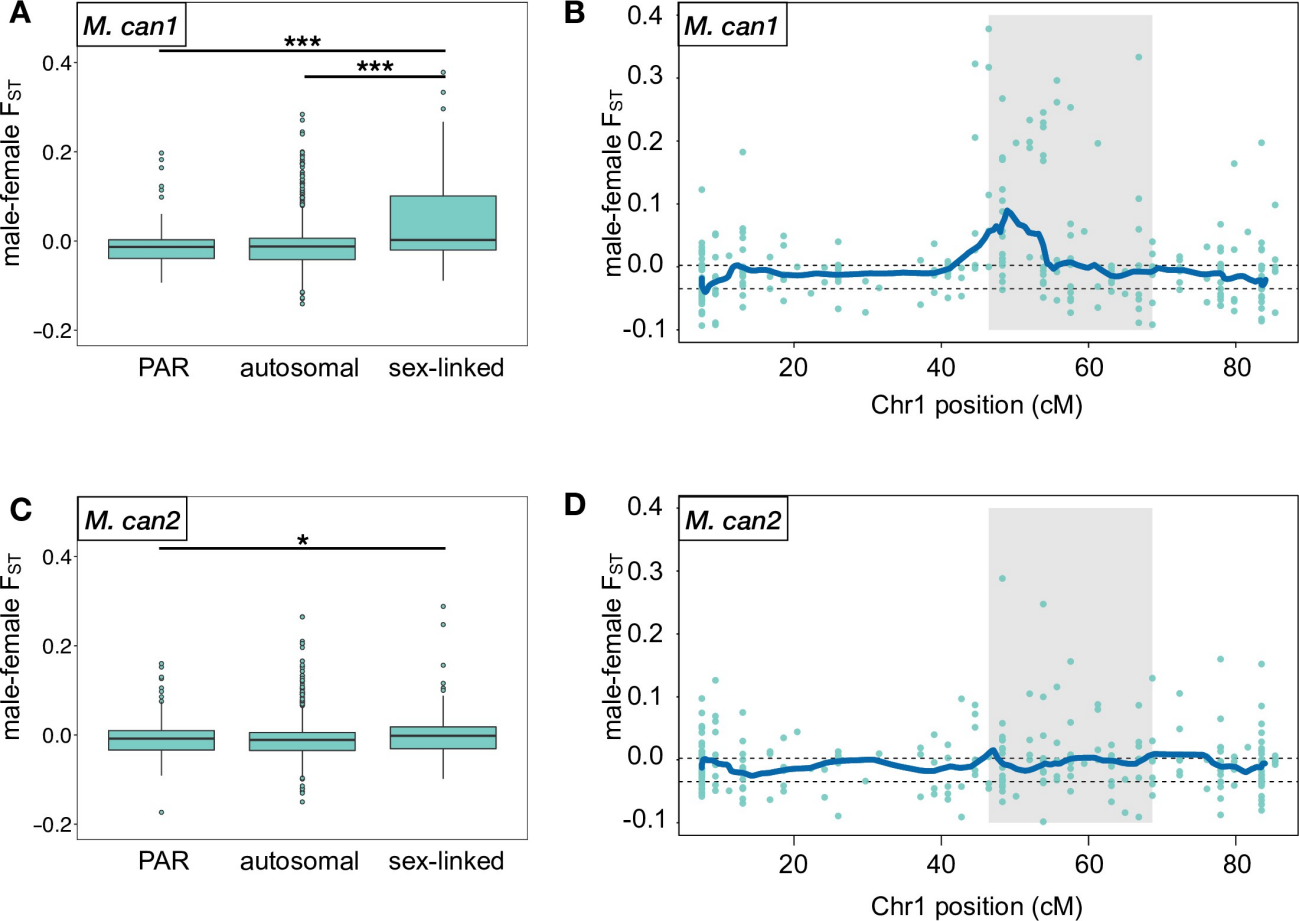
*F*_ST_ between males and females in *M*. *canariensis*. (A) Boxplot of *F*_ST_ between males and females in the *M*. *can1* subgenome, with autosomal, pseudoautosomal, and sex-linked categories defined according to the linkage map for *M*. *annua* [60]. (B). Rolling average of 20 transcripts across linkage group 1 in the *M*. *can1* subgenome. Grey shaded regions indicate the sex-linked region inferred for *M*. *annua* [60]. Horizontal dashed lines show 95% CI based on comparison with autosomes. (C). Boxplot of *F*_ST_ between males and females in autosomal, pseudoautosomal, and sex-linked regions in *M*. *can2* subgenome. (D) Rolling average of 20 transcripts across linkage group 1 in *M*. *can2* subgenome.

To detect sex-linked regions in the *M*. *canariensis* genome, we computed *F*_ST_ between males and females for each transcript. As a first step, we categorized each transcript as sex-linked, autosomal, or pseudoautosomal (i.e., in the recombining pseudoautosomal region, PAR), again based on the *M*. *annua* linkage map [60]. To test the hypothesis that *M*. *canariensis* inherited its sex-linked region from *M*. *annua*, we compared the sex-linked regions to the PAR and autosomes within each subgenome. We found that *F*_ST_ between males and females was elevated in the sex-linked region relative to the PAR and the autosomes in the *M*. *can1* subgenome (Fig 3A; sex-linked vs. PAR, Wilcoxon test: p = 4.80 x 10^−6^; sex-linked vs. autosomes: p = 1.70 x 10^−7^). In the *M*. *can2* subgenome, by contrast, there was no difference between the sex-linked region and the PAR (Fig 3C; Wilcoxon test: p = 0.2351), and *F*_ST_ in the sex-linked region was elevated relative to the autosomes, but the effect was much weaker than in the *M*. *can1* subgenome and likely due to residual mapping of reads derived from *M*. *can1* (Fig 3C; Wilcoxon test: p = 0.02998). Moreover, there was no significant difference between the autosomes or PAR in either subgenome (Fig 3A; Wilcoxon test: p = 0.9191, Fig 3C; Wilcoxon test: p = 0.2917), and no regions of elevated *F*_ST_ were detected on the autosomes (S4 Fig). This pattern implies that the sex-determining region is in the *M*. *can1* subgenome and thus derived directly from a recent ancestor of extant *M*. *annua*.

To locate the sex-determining region more precisely, we computed *F*_ST_ in a sliding window. We calculated *F*_ST_ for each transcript and computed a rolling average of *F*_ST_ for 20 consecutive transcripts. To compute 95% confidence intervals, we similarly computed the rolling average for 20 consecutive transcripts on autosomes 1,000 times (linkage groups 2 to 8 in *M*. *can1*, i.e., excluding the putative sex chromosome; all linkage groups of *M*. *can2*). This identified a single region of elevated *F*_ST_ corresponding to linkage group 1 of the *M*. *can1* subgenome (Fig 3B). This region largely overlapped with the sex-determining region identified in *M*. *annua* [60]. We detected no other regions of elevated *F*_ST_ elsewhere in the *M*. *can1* or *M*. *can2* subgenomes (Figs 3D and S4).

### No evidence of subgenome dominance

To evaluate the possibility of preferential gene loss or inactivation in one subgenome over the other, we aligned our short reads from *M*. *canariensis* to the *M*. *annua* transcriptome. Both members of the homeologous pair should align to their ortholog in *M*. *annua*, resulting in increased within-individual heterozygosity when both members of the pair are present relative to when only one homeolog is present or expressed. It is worth noting that reads derived from the *M*. *can2* subgenome are expected to map less efficiently than reads derived from the *M*. *can1* subgenome, potentially leading to an underestimate of heterozygosity. The distribution of heterozygosity (measured in terms of within-individual SNP density, S5 Fig) shows that the majority of genes are highly heterozygous, as expected for a polyploid genome. A secondary smaller peak is found at low heterozygosity (consisting almost exclusively of genes for which only one transcript was found in the assembly), arguing against widespread gene loss. Since *M*. *can1* is less diverged from *M*. *annua* than *M*. *can2*, divergence with *M*. *annua* should be lower when *M*. *can1* is present than when *M*. *can2* is maintained instead. We therefore computed the average SNP density relative to *M*. *annua* as a proxy for divergence. Putative single-copy genes (heterozygosity<0.01) had a wide range of divergence levels, suggesting that gene loss has not occurred primarily in one of the two subgenomes (S5 Fig).

We then asked whether the strength of purifying selection differed between *M*. *can1* and *M*. *can2*. We computed Tajima’s D and π_N_/π_S_ for each transcript in both subgenomes. Tajima’s D is lower in *M*. *can2* than *M*. *can1* for all eight linkage groups (S6 Fig; paired Wilcoxon test, p = 0.007813). The fact that Tajima’s D is low across the whole genome points to demographic fluctuations rather than selection as responsible for distorting the coalescent branch lengths. Furthermore, π_N_/π_S_ did not differ between the two subgenomes (S7 Fig; paired Wilcoxon test, p = 0.3828). All measures of π (π, π_N_, and π_S_) are elevated in *M*. *can1* compared to *M*. *can2* (S8–S10 Figs; paired Wilcoxon test: p = 0.00781 for all three measures). *M*. *can1* is thus generally more polymorphic than *M*. *can2*, likely because there was a greater level of ancestral polymorphism in the *M*. *annua*-like ancestor of *M*. *can1* than the (unknown) ancestor of *M*. *can2*. We therefore conclude that there is no evidence of different levels of purifying selection between the two subgenomes.

Finally, we characterized expression for each transcript in the reduced *M*. *canariensis* transcriptome. In our analyses, we considered all genes assigned to a subgenome, regardless of whether or not they can be assigned to a linkage group location based on the *M*. *annua* linkage map. Of the 2,427 pairs examined, nearly half (1,018) had significantly different expression between the two subgenomes in females, and approximately 1/3 (880 genes) were expressed at different levels in males. However, in both sexes the direction in subgenome bias was approximately equal, with 515 expressed more highly in *M*. *can1* and 503 expressed more highly in *M*. *can2* in females and 447 expressed more highly in *M*. *can1* and 433 expressed more highly in *M*. *can2* in males (S11 Fig). We therefore find no evidence for subgenome dominance in *M*. *canariensis*.

### Low levels of sex-biased gene expression and sexual subfunctionalization

We detected 12,918 genes expressed in mature leaves; of these, 80 (0.6%) were sex-biased, with 46 and 34 male- and female-biased, respectively (S12 Fig). We could only assign 17 of the 80 sex-biased genes to linkage groups, and only 10 to linkage groups in the *M*. *can1* subgenome. However, four of the ten sex-biased genes (40%) in the *M*. *can1* subgenome were located on linkage group 1, a proportion that is substantially greater than the 17% (262/1,500) of all expressed genes on linkage group 1 in the *M*. *can1* subgenome. However, although this trend is similar to that found for diploid *M*. *annua* [62], it falls short of statistical significance (Fisher’s exact test, p = 0.0807).

We asked whether homeologous pairs show evidence of sexual subfunctionalization. Sexual subfunctionalization could consist of individual homeologous pairs, with one member being male-biased and the other being female-biased. Alternatively, only one member of a homeologous pair may evolve sex-bias, while the other may retain a sex-independent function [26]. Finally, it is also possible that both members of the homeologous pair are sex-biased in the same direction, with one more so than the other.

**Fig 4:**
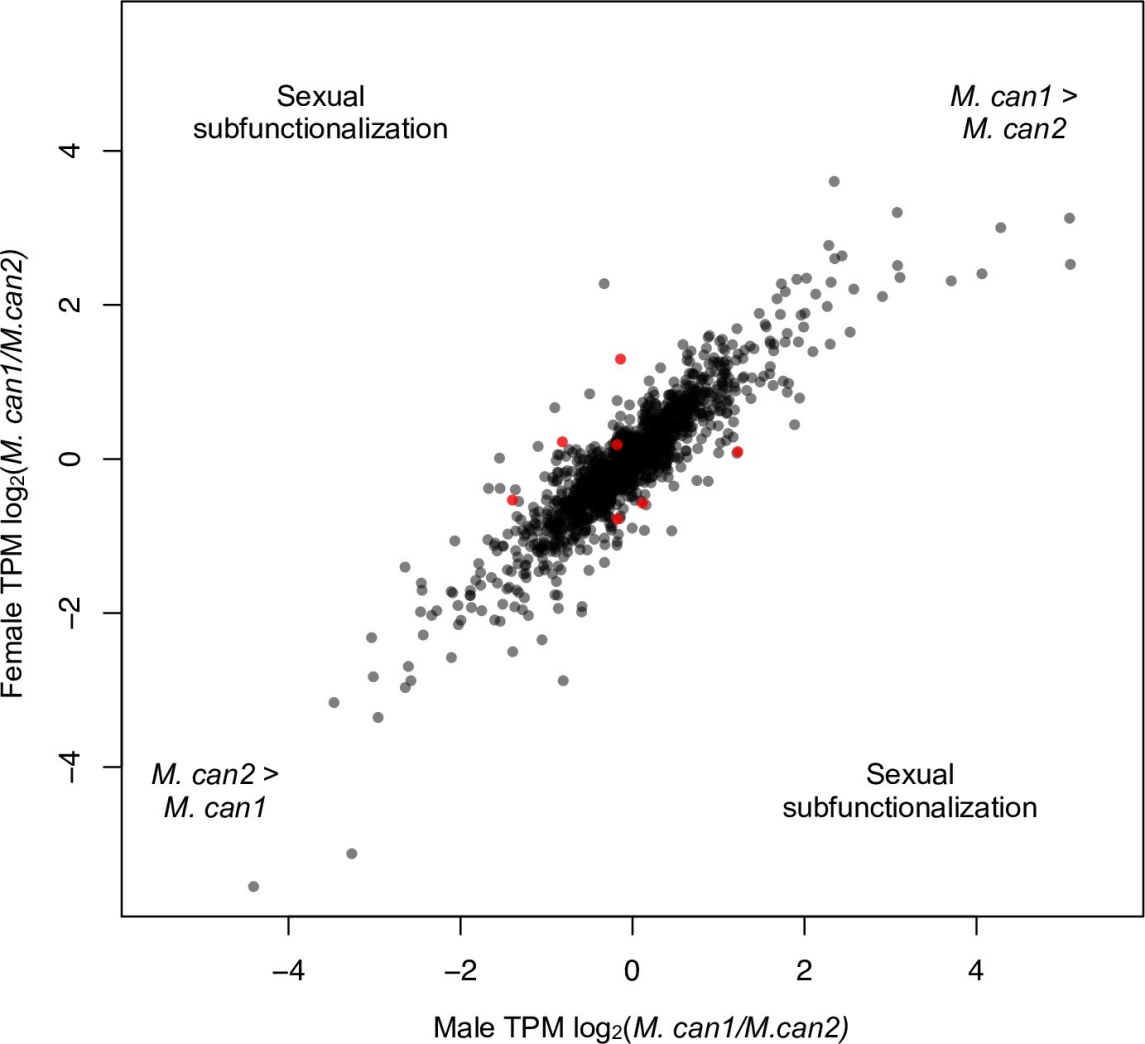
Potential subfunctionalization of *M*. *can1* and *M*. *can2* subgenomes. Expression is shown in TPM (transcripts per million). Several homeolog pairs are subfunctionalized (expressed higher in one subgenome). Seven pairs of homeologs showed evidence of sexual subfunctionalization (shown in red).

To identify genes with the most divergent sex bias between the homeolog pairs, we computed the log_2_(TPM *M*. *can1*/TPM *M*. *can2*) for each individual. We then compared these ratios between males and females using the “robust” option in the R package Limma [63]. We identified seven genes showing evidence of sexual subfunctionalization (Fig 4). We found a similar number of genes that were female-biased in *M*. *can1* and male-biased in *M*. *can2* (3) as the reverse (3), suggesting that sexual subfunctionalization evolves independently within a given homeologous pair. Additionally, we detected one gene that was more male-biased in *M*. *can1* than *M*. *can2*. While we only identify seven genes with evidence of sexual subfunctionalization, two of these genes were located in the SDR and were more female-biased in *M*. *can1* than *M*. *can2*. While this result suggests that the SDR of *M*. *can1* is enriched for sexually subfunctionalized genes, it falls short of statistical significance (Fisher’s exact test, p = 0.05742). Importantly, these results suggests that genes on the Y chromosome may be undergoing degeneration on a gene-by-gene basis, but we do not detect a large-scale pattern of Y degeneration.

## Discussion

### Origin of allopolyploid *M*. *canariensis* and its sex-determining region

Our results indicate that the diploid *M*. *annua* lineage was one of the two progenitors of allopolyploid *M*. *canariensis*, confirming the conclusions of Obbard et al. [55]. Further, *M*. *canariensis* inherited its sex-determining region (SDR) directly from *M*. *annua* (see S13 Fig for a more detailed hypothesis of *M*. *canariensis* formation). The SDR of *M*. *canariensis* spans a region from approximately 40 cM to 55 cM on linkage group 1 of the *M*. *annua* linkage map [60]. As in the sex-determining region of *M*. *annua* [60], divergence between the X and Y chromosomes in this region in *M*. *canariensis* is low, with no deficit of heterozygosity in males (S14 Fig). Interestingly, the inferred SDR for *M*. *canariensis* only partially overlaps with that inferred for *M*. *annua* ([60]; Fig 3B), suggesting that the *M*. *annua* SDR may have expanded since the allopolyploid origin of *M*. *canariensis*. This interpretation is consistent with the previous identification of two strata on the sex chromosomes of *M*. *annua*, the younger of which emerged after *M*. *annua* diverged from its sister species, *M*. *huetii*, approximately a million years ago [60], though that study was unable to locate these strata precisely using the *M*. *annua* linkage map. Given that the SDR of *M*. *canariensis* overlaps with that of *M*. *annua* in the region of 46 cM to 55 cM (based on the corresponding female linkage map of *M*. *annua*; [60]), this region might harbor the sex-determining locus of all three species and have stopped recombination earliest. Finally, we note that the SDR of *M*. *canariensis* might have expanded in the opposite direction to that of *M*. *annua* after its allopolyploid origin (i.e., towards ~ 40 cM), although evidence for this possibility is much weaker.

### Subgenome dominance and sexual subfunctionalization

We could detect no evidence for subgenome dominance in gene loss, purifying selection, or gene expression in mature leaf tissues of *M*. *canariensis*, in contrast to findings for other polyploid species (e.g.,[64–66]. It is possible that genome dominance is absent in *M*. *canariensis* because the two progenitor species were not sufficiently highly diverged, with little difference in the content and distribution of transposable elements at the time of polyploidization, which is thought to be its basis [65,67,68]. It is also possible that *M*. *canariensis* originated so recently that gene loss and silencing have simply not had time to establish a dominant subgenome. To better understand the timing of these events, we estimated the TMRCA of *M*. *can1* and *M*. *annua*, and *M*. *can1* and *M*. *can2*. Using a mutation rate of 7.5 x 10^−9^ [60], we estimated that the two subgenomes diverged ~9.4 million generations ago, and that *M*. *can1* and *M*. *annua* diverged ~4.7 million generations ago. Finally, subgenome dominance may indeed have evolved in *M*. *canariensis*, though in tissues other than the mature leaves that we sampled, as has been found, for instance, in allotetraploid blueberry [69] and cotton [65].

The small proportion of sex-biased genes that we observed in mature leaves of *M*. *canariensis* was similar to that found for diploid *M*. *annua* (~2%; [62]) as well as the vegetative tissues of several other plant species including *Populus tremula* [70], *Populus balsamifera* [71], and *Salix viminalis* [72]. However, in diploid *M*. *annua*, sex-biased genes were three times more likely to be female-biased than male-biased, whereas in *M*. *canariensis* we found slightly more male-biased genes. Sex-biased genes detected in vegetative tissues are overrepresented on the sex chromosomes of *M*. *annua* [62]. Similarly, examination of gene expression reproductive tissues has detected an enrichment of sex-biased genes in the sex-linked region in *Asparagus officinalis* [73], *Silene latifolia* [74], and *Cannabis sativa* [75], but not in *Populus balsamifera* [71] or *Salix viminalis* [72]. We also detected an overrepresentation of sex-biased genes on the sex chromosomes, though the difference was not statistically significant.

The differences in sex-biased gene expression between two closely related species that share a genome contribute to an emerging picture of rapid change in gene expression over time, and between different expression contexts [76]. In the case of *Mercurialis*, Cossard et al. [62] found little correspondence between the identity of male- or female-biased genes even between different stages of leaf development in diploid *M*. *annua* separated by a few days in early seedling growth. Thus, while sex-biased gene expression in plants is of substantial interest for understanding the evolution of sexual dimorphism [71–74,77,78], and indeed the evolution of gene expression itself in different genetic and phenotypic contexts, interpretations of its importance and meaning ultimately need to be articulated in the context of these apparent vicissitudes in expression.

We found that seven gene pairs that showed evidence of sexual subfunctionalization in *M*. *canariensis*, i.e., a divergence in expression between the two homeologous gene pairs that corresponds to male versus female functions. Thus, in our analysis, sexual subfunctionalization in *M*. *canariensis* was limited to a few genes in vegetative tissues. However, we examined only a small proportion of the genome (1503 homeologous pairs out of an estimated 21,000), so our power to detect sexual subfunctionalization was limited. Nevertheless, sexual dimorphism in gene expression tends to be greater in reproductive tissues than vegetative tissues in plants [71,72], so it seems possible that sexual subfunctionalization may have evolved for more genes in *M*. *canariensis* that are expressed in sexually dimorphic reproductive tissue, e.g., flower buds.

### Evolutionary history of dioecy in *Mercurialis*

Our study throws further light on the role of allopolyploidy in the diversification of *Mercurialis*. Obbard et al.[55] hypothesized that the diploid *M*. *annua* was a progenitor of *M*. *canariensis* on the basis of phylogenetic inference using ITS and chloroplast DNA sequences. In addition, both tetraploid *M*. *annua*, a monoecious lineage currently distributed in central western Morocco, and the two hexaploid lineages currently distributed in the Iberian Peninsula [79] also have ITS and chloroplast sequences that point to an ancestor of diploid *M*. *annua* as a progenitor [55]. It is now clear that an ancestor of diploid *M*. *annua* has been a progenitor for more than one polyploid species, with two or more independent origins. Polyploidy is thought to be polyphyletic in other plant clades too, with ploidal races comprising populations with more than one independent origin, involving the same progenitor or progenitors [79–81]. In other cases, the same diploid progenitor has contributed to the polyploid origin of more than one species by hybridizing with more than one other diploid species [81,82]. For instance, the diploid plant *Tragopogon dubius* was inferred to have been a progenitor of both *T*. *mirus* and *T*. *miscellus* via allopolyploid hybridization with *T*. *porrifolius* and *T*. *pratensis*, respectively [83]. *M*. *annua* provides a further such example, with no fewer than three distinct lineages now carrying and expressing its genome.

As just noted, the allotetraploid and allohexaploid lineage of *M*. *annua* to which diploid *M*. *annua* has contributed its genome are composed largely of monoecious individuals. Perhaps significantly, the early descendants of artificial neo-polyploids of *M*. *annua*, as reported by Durand [54], are also monoecious, suggesting that polyploidization might have played a role in the breakdown of dioecy in polyploid *M*. *annua*, at least initially. If this is a general feature of the effect of genome duplication in the genus [30,31], then the fact that *M*. *canariensis* is dioecious would indicate that dioecy re-evolved subsequent to its loss. In part of the range of hexaploid *M*. *annua*, androdioecy appears to have evolved back to subdioecy, with the monoecious individuals producing almost no male flowers (thus being functionally female), and population sex ratios being close to 1:1 [84]. Such a process might have given rise to dioecy in *M*. *canariensis*, too. Obbard et al. [55] hypothesized that the male-determining allele in androdioecious hexaploid *M*. *annua* might have been donated by its other putative diploid progenitor, *M*. *huetii*, because its plastid genome evidently came from *M*. *annua*. Verification of that scenario awaits comparison of the hexaploid *M*. *annua* Y chromosome with the Y chromosomes of *M*. *huetii*. In contrast, because our study here involved characterization of the sex-determining region of *M*. *canariensis*, it is immediately possible to infer not only that *M*. *annua* was one of its two diploid progenitors, but also that *M*. *annua* is the origin of the *M*. *canariensis* sex-determining locus. Thus, whether or not dioecy in *M*. *canariensis* evolved via androdioecy, as might be the case for the subdioecious populations of hexaploid *M*. *annua* in Morocco, it seems that its dioecious sexual system did not originate in either lineage with the evolution of a completely new sex-determining locus.

Our results also suggest that the same sex-determining system is likely shared across all the annual *Mercuries* lineages with separate sexes. Previous work by Russell and Pannell [56] used interspecific crosses to show that the underlying architecture of sex determination has been maintained throughout the *M*. *annua* and *M*. *huetii* species complex. Interspecific crosses of males from diploid *M*. *annua*, *M*. *huetii*, or hexaploid androdioecious *M*. *annua* with hermaphrodites or females of any of these species (including tetraploid *M*. *annua*) produced ~ 50% male offspring, suggesting that the Y chromosome in males of *Mercurialis* retain their dominance over the recessive X chromosome locus in females and hermaphrodites [56]. Although Russell and Pannell [56] did not include *M*. *canariensis* in their crossing scheme, our study suggests that they would have had similar results with it, too.

### Implications for our understanding of ploidy and sexual-system evolution

Polyploidization is a process that can involve a disruption of meiotic segregation, gene expression, the proliferation of transposable elements, and epigenetic marking [8,30,85]. When it involves dioecious species, polyploidization might also interfere with sex determination, precipitating the breakdown of dioecy and the reversion to hermaphroditism [30], as was found in artificially generated tetraploids of *M*. *annua* cited above (Durand 1963). However, our study demonstrates the inheritance of an intact sex-determining region in an allopolyploid from a diploid progenitor. Although this a possibility was implied by the results Obbard et al [55] and Russell and Pannell [56], our results here represent the first more direct indication on the basis of genome analysis for the retention of dioecy through allopolyploidization.

Gene duplication has been shown to resolve sexual antagonism in a number of diploid taxa by breaking sexual correlations due to pleiotropic constraints [26–28]. Our study extends this idea to homeologous pairs in a polyploid plant. While we only found evidence for sexual subfunctionalization for a few genes, it may also have played a role in the evolution of gene expression in reproductive tissues in *M*. *canariensis*, which we did not sample, and may be important in other polyploid species. In this sense, we believe that sexual subfunctionalization may be an underappreciated possibility in evolution of dioecy in polyploids, including those derived from hermaphroditic diploids. It is of course also possible that sexual subfunctionalization may be an alternative route to subgenome dominance in dioecious polyploids, though this does not yet appear to have been examined in any other species.

Finally, we note that *M*. *canariensis* is the only known polyploid *Mercurialis* species extant on an island. Dioecy is more common in island taxa than their relatives on the mainland [86–91], a fact that may appear surprising, given that self-compatible hermaphrodites are thought to be able to colonize islands more easily than self-incompatible hermaphrodites or dioecious species [92,93]. It is possible that the higher frequency of dioecy on oceanic islands is due to the evolution of separate sexes after colonization [92]. It is often difficult to know whether a dioecious island species evolved separate sexes prior to colonization or afterwards, although phylogenetic information can be helpful [88]. In the case of *M*. *canariensis*, it seems likely that dioecy evolved prior to colonization, given that at least one of its progenitors, *M*. *annua*, is a widespread continental species. If so, either a male and a female colonized together or, more likely perhaps, the island was colonized by a ‘leaky’ male that produced one or a few female flowers. Such leakiness in sex expression is common in diploid *M*. *annua* [94,95] and may have been present in the colonizing ancestors of extant *M*. *canariensis*.

## Materials & methods

### Study material

We sampled seeds of *M*. *canariensis* from six populations on the island of Tenerife. Six males and seven females were grown together in the greenhouse at the University of Lausanne in the spring of 2017. For each individual, we extracted RNA from 10 mature leaves using the Qiagen plant RNAeasy kit. For six males and six females, individual libraries were prepared using the TruSeq Stranded mRNA Sample Prep Kit and sequenced on the Illumina HiSeq2000. The remaining female was sequenced on a PacBio SMRT Cell on the Sequel I platform. All samples were sequenced at the Center of Integrative Genomics at the University of Lausanne.

### Transcriptome and quality control

The Center of Integrative Genomics processed the PacBio sequencing data using the long-read isoform sequencing pipeline, IsoSeq3. This sidesteps the problem of assembling transcripts from short reads by producing full-length transcripts. This resulted in a transcriptome assembly consisting of 24,375 transcripts. ORFs were extracted using Transdecoder v.5.5 [96].

We assessed sequence quality of our short reads using FASTQC v.0.11.6 (www.bioinformatics.babraham.ac.uk/projects/fastqc). Adapter removal and trimming used Trimmomatic v.0.36 [97], with options ILLUMINACLIP: TruSeq3-PE.fa:2:30:10 LEADING:3 TRAILING:3 SLIDINGWINDOW: 4:15 MINLEN:36. After filtering, we retained an average of 44.7 million paired-end sequences per sample.

### Homeolog identification and genomic location

To identify homeologs with confidence, we first removed all redundant transcripts and isoforms from our IsoSeq assembly using a custom perl script. We first ran an all-vs-all blat within the IsoSeq3 assembly and identified transcripts with high sequence identity. We grouped transcripts together that had a match score > 50 and divergence of < 1%, and retained only the longest transcript. We also implemented this pipeline on the published transcriptome of *M*. *annua*.

We then used our reduced assemblies to identify homeologs with *M*. *canariensis*. We again implemented an all-vs-all blat within the *M*. *canariensis* transcriptome. We selected transcript pairs in which the second-best hits were reciprocal (as the best hit is the ‘self’). These were preliminarily assigned as homeolog pairs. In parallel, for each *M*. *canariensis* transcript, we used blat to identify the best hit in the nonredundant *M*. *annua* transcriptome. We required putative homeolog pairs to have the same best blat hit in the *M*. *annua* transcriptome.

For each homeolog pair, we calculated dS with the best-hit *M*. *annua* transcript using KaKs_calculator (http://code.google.com/p/kaks-calculator/). Within each pair, the transcript with lower dS was preliminarily assigned to the *M*. *can1* subgenome (Fi 1A; putatively derived from M. *annua*) and the higher dS was assigned to *M*. *can2* (putatively derived from an unknown progenitor species). Furthermore, we used Ka/Ks calculator (http://code.google.com/p/kaks-calculator/) to compute dS between homeologs.

To ensure that our homeolog pairs were indeed homeologs, as opposed to isoforms or alleles of the same gene, we constructed gene trees. We included both members of the homeolog pair, their ortholog in *M*. *annua*, and the reciprocal best-hit match between *M*. *annua* and *Ricinus communis*. We constructed gene trees with default parameters and the GTRGAMMA model of nucleotide evolution in RAxML [98], and rooted trees using Treebender in the Phylommand software package [99]. To visualize the resulting gene trees, we constructed a cloudogram using Densitree v.2.2.27 [100]. Homeologs were considered correctly assigned if the gene trees had the expected structure of (((*M*. *can1*, *M*. *annua*), *M*. *can2*), *R*. *communis*).

We used linkage map information from *M*. *annua* [60] to assign each homeolog pair to linkage groups. Veltsos et al. [60] assigned a total of 8490 transcripts to linkage groups, however some of these transcripts were removed in the nonredundant transcriptome. In total, 8181 nonredundant transcripts were assigned linkage group positions in *M*. *annua*. We parsed each pair into the *M*. *can1* and *M*. *can2* subgenomes based on their dS values and gene tree structure.

### Identifying the sex-determining region in *M*. *canariensis*

To identify the sex-determining region, we evaluated allele frequency differences between males and females by computing *F*_ST_ [101–103]. In order to maximize the number of reads that map to each homeolog, we kept only the longest isoform per gene in all subsequent analyses, and used the exon-junction aware software STAR v.2.6 [104] for the alignment. This allows us to incorporate all read information when identifying the sex-determining region of *M*. *canariensis*. Further, it allows us to compare sex-biased gene expression, homeolog-biased gene expression, and sexual subfunctionalization of genes with confidence. We then processed the alignments with Picard Tools v.1.141 (http://broadinstitute.github.io/picard/). We ran Samtools v.1.11 mpileup [105] with the probabilistic alignment disabled, and called SNPs using Varscan v2.4.3 [106], with a minimum variant allele frequency of 0.15, and a minimum threshold for homozygotes of 0.85. We used VCFtools v.0.1.16 [107] to require a minimum of 10 reads per site for all individuals and a Phred quality score > 20. We then computed *F*_ST_ for each transcript over 100 bp using VCFtools v.0.1.16 [107].

As a second measure, we computed sex-biased heterozygosity for each transcript following the method outlined in Toups et al. [108]. We defined heterozygosity as the fraction of heterozygous sites per transcript, and we then computed the log_10_ ratio of male heterozygosity:female heterozygosity. This measure of sex-biased heterozygosity should be ~ 0 for autosomal transcripts. For sex chromosomes, sex-biased heterozygosity should be positive in less differentiated XY systems where males have an excess of heterozygous sites, and negative in highly differentiated XY systems due to hemizygosity in males.

We defined sex-linked, autosomal, and pseudoautosomal (PAR) regions of the two subgenomes of *M*. *canariensis* according to the *M*. *annua* linkage map [60] and assessed the significance between these regions using Wilcoxon signed rank tests. We computed moving averages in sliding windows of 20 transcripts on each linkage group using the rollmean function from the package zoo in R v.4.0.3. To identify regions of elevated FST and sex-biased heterozygosity, we computed 95% confidence intervals by sampling rolling averages of 20 consecutive autosomal transcripts 1000 times.

### Expression analysis

To assess gene expression, we generated pseudoalignments of our reads to the reduced *M*. *canariensis* transcriptome using Kallisto v.0.43.1 [109]. We used the tximport package [110] to import transcript-level abundance, estimated counts, and transcript lengths for downstream analyses in DEseq2 [111]. As a first step, we analysed sex-biased gene expression for each *M*. *canariensis* transcript using count matrices. As a second step, we assessed differential expression of homeologs between subgenomes. In both analyses, we corrected p-values for multiple tests using Benjamini and Hochberg’s algorithm [112].

For our sexual subfunctionalization analysis, we converted our count information to TPM, and then computed the ratio of log_2_(*M*. *can1* TPM/*M*. *can2* TPM) for each sample. We then constructed a linear model for each gene pair in the R package limma [63] using the “robust” option. We corrected for multiple tests using the Benjamini and Hochberg algorithm [112], and identified significant gene pairs using an adjusted p-value of <0.05.

### Computing measures of selection

To determine if one subgenome was under stronger purifying selection than the other, we computed two measures of selection, Tajima’s D and π_N_/π_S_, for each transcript. In addition, to better understand the differing demographic effects on the two subgenomes, we also calculated π, π_N_, and π_S_. Tajima’s D and pi were calculated in VCFTools v.0.1.16 [107] and π_N_, π_A_, and π_N_/π_S_ were calculated using the PopPhyl pipeline [113]. To eliminate the effects of linkage, we computed the mean metric for each chromosome and compared the two subgenomes using paired Wilcoxon tests.

## Supporting information

S1 Fig Pipeline to assign homeologs to the *M*. *can1* and *M*. *can2* subgenomes. After assembly of long-read PacBio sequencing using the Isoseq3 pipeline, an all-vs-all blat was used to form clusters of highly similar sequences and only the longest isoform per cluster was retained in the transcriptome. A second all-vs-all blat was performed on the transcriptome consisting of the longest isoforms, and this was used to locate reciprocal best hits. These reciprocal best hits were treated as potential homeolog pairs. We then used blat to identify the closest sequence for each homeolog in the *M*. *annua* transcriptome, and filtered only for homeolog pairs that blat to the same *M*. *annua* transcript. Finally, we calculated dS between each member of a retained homeolog pair and *M*. *annua*, and the homeolog with lower dS was assigned to the *M*. *can1* subgenome (putatively derived from P1 –the same lineage as *M*. *annua*) and the other homeolog was assigned to the *M*. *can2* subgenome (putatively derived from P2, the unknown progenitor).
(TIF)

S2 Fig *F*_ST_ between males and females in *M*. *canariensis* using only gene trees to assign homeologs to subgenomes. (A) Boxplot of *F*_ST_ between males and females in the *M*. *can1* subgenome, with autosomal, pseudoautosomal, and sex-linked categories defined according to the linkage map for *M*. *annua*. (B) Rolling average of 20 transcripts across linkage group 1 in the *M*. *can1* subgenome. Grey shaded regions indicate the sex-linked region inferred for *M*. *annua*. Horizontal dashed lines show 95% CI based on comparison with autosomes. (C) Boxplot of *F*_ST_ between males and females in autosomal, pseudoautosomal, and sex-linked regions in the *M*. *can2* subgenome. (D) Rolling average of 20 transcripts across linkage group 1 in the *M*. *can2* subgenome.
(TIF)

S3 Fig Potential subfunctionalization of the *M*. *can1* and *M*. *can2* subgenomes, where homeologs are assigned to subgenome using only gene trees. Expression is shown in TPM (transcripts per million). Several homeolog pairs are subfunctionalized (expressed higher in one subgenome). Six pairs of homeologs showed evidence of sexual subfunctionalization (shown in red).
(TIF)

S4 Fig FST on autosomes. FST in rolling windows of 20 transcripts across linkage groups 2–8 in both the *M*. *can1* and *M*. *can2* subgenomes. Horizontal dashed lines show 95% CI.
(TIF)

S5 Fig Within-individual heterozygosity when aligned to *M*. *annua* vs. SNP divergence from *M*. *annua*. Blue triangles show values for transcripts in which both homeologs are present, whereas green circles show values when no homeolog is identified for a transcript. We hypothesized that when only the *M*. *can1* homeolog was present, within-individual heterozygosity and divergence from *M*. *annua* should be low, while when only the *M*. *can2* homeolog was present, within-individual heterozygosity should be low while divergence with *M*. *annua* should be high. However, because there are no two distinct distributions in the divergence from *M*. *annua*, it is not possible ¨to assign single homeologs to subgenomes.
(TIF)

S6 Fig Tajima’s D for both the *M*. *can1* and *M*. *can2* subgenomes across the eight linkage groups. (TIF)

S7 Fig Π_N_/π_s_ for both the *M*. *can1* and *M*. *can2* subgenomes across the eight linkage groups. (TIF)

S8 Fig Π for both the *M*. *can1* and *M*. *can2* subgenomes across the eight linkage groups. (TIF)

S9 Fig Π_N_ for both the *M*. *can1* and *M*. *can2* subgenomes across the eight linkage groups. (TIF)

S10 Fig Π_s_ for both the *M*. *can1* and *M*. *can2* subgenomes across the eight linkage groups. (TIF)

S11 Fig Differentially expressed genes between the *M*. *can1* and *M*. *can2* subgenomes in (A) females and (B) males. Genes that are significantly differently expressed between subgenomes are shown in red.
(TIF)

S12 Fig Differentially expressed genes between females and males in (A) the *M*. *can1* and (B) the *M*. *can2* subgenomes. Genes that are significantly differently expressed between the sexes are shown in red.
(TIF)

S13 Fig Hypothesis for how hybridization and polyploidization gave rise to *M*. *canariensis*. *M*. *canariensis* most likely formed via a triploid bridge, which requires at least two hybridization events: one resulting in males and one resulting in females. Males most likely evolved when an unreduced gamete from diploid, male *M*. *canariensis* fused with a haploid gamete containing the X chromosome of the unknown progenitor species. This resulted in a triploid offspring, which backcrossed with a haploid gamete containing the X chromosome from the unknown progenitor species. Females also likely formed via a triploid bridge pathway, though there are at least three possible crossing scenarios that result in the XXXX genotype. Figure adapted from [114].
(TIF)

S14 Fig Sex-biased heterozygosity (SBH: ratio of male:female heterozygosity) in *M*. *canariensis*. (A) Boxplot of SBH between males and females in the *M*. *can1* subgenome, with autosomal, pseudoautosomal, and sex-linked categories defined according to the linkage map for *M*. *annua* [60]. (B) Rolling average of 20 transcripts across linkage group 1 in the *M*. *can1* subgenome. The grey rectangle indicate the sex-linked region in *M*. *annua* [60]. Horizontal dashed lines show 95% CI based on comparison with autosomes. (C) Boxplot of SBH between males and females in autosomal, pseudoautosomal, and sex-linked regions in the *M*. *can2* subgenome. (D) Rolling average of 20 transcripts across linkage group 1 in the *M*. *can2* subgenome.
(TIF)
