# Supplementary figures and images for: Dioecy and chromosomal sex determination are maintained through allopolyploid speciation in the plant genus Mercurialis
Source: PLoS Genet. 2022 Jul 6;18(7):e1010226. doi: 10.1371/journal.pgen.1010226 (PMC9292114; doi:10.1371/journal.pgen.1010226)

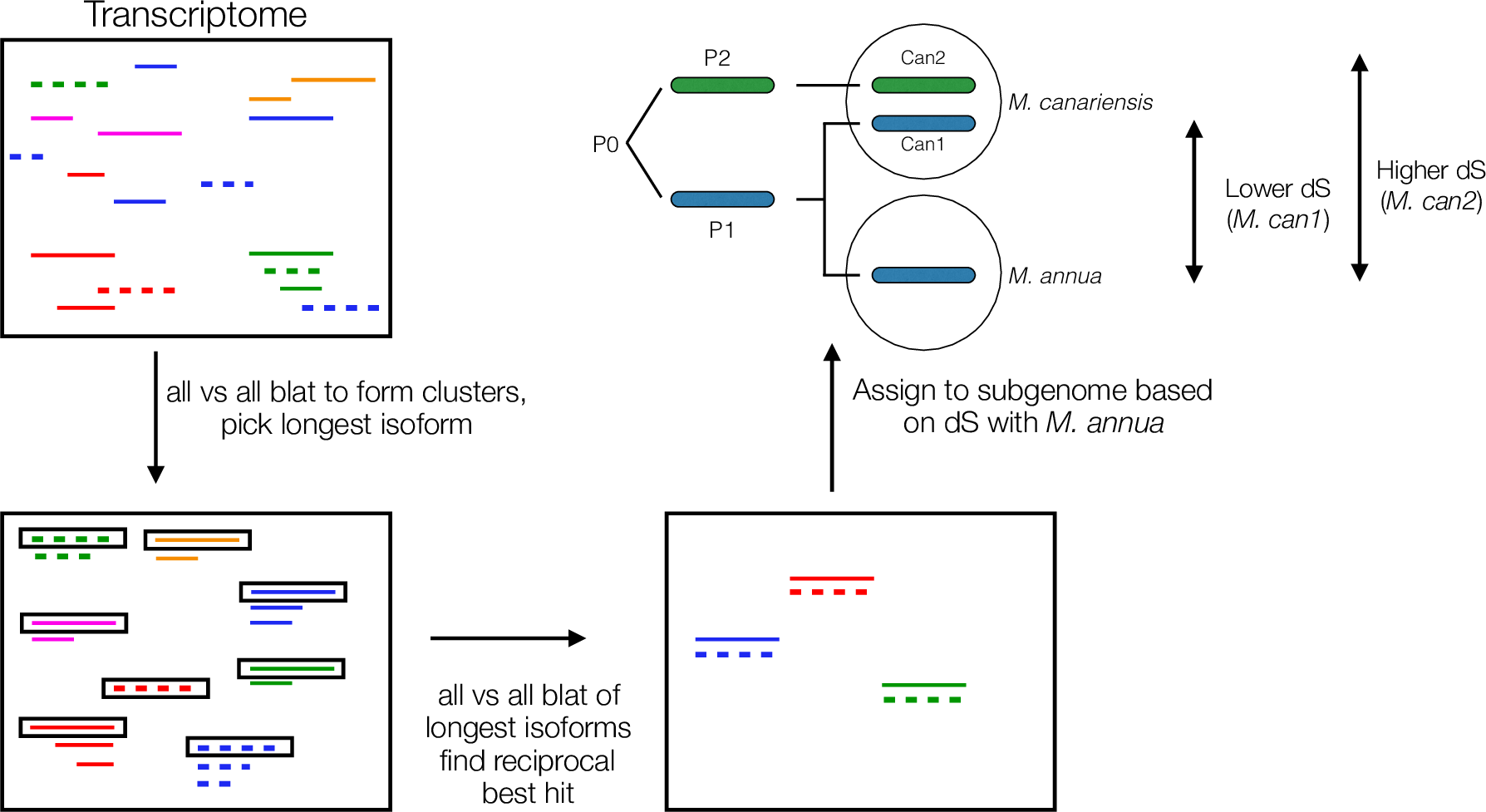

Supplement: S1 Fig — After assembly of long-read PacBio sequencing using the Isoseq3 pipeline, an all-vs-all blat was used to form clusters of highly similar sequences and only the longest isoform per cluster was retained in the transcriptome. A second all-vs-all blat was performed on the transcriptome consisting of the longest isoforms, and this was used to locate reciprocal best hits. These reciprocal best hits were treated as potential homeolog pairs. We then used blat to identify the closest sequence for each homeolog in the M. annua transcriptome, and filtered only for homeolog pairs that blat to the same M. annua transcript. Finally, we calculated dS between each member of a retained homeolog pair and M. annua, and the homeolog with lower dS was assigned to the M. can1 subgenome (putatively derived from P1 –the same lineage as M. annua) and the other homeolog was assigned to the M. can2 subgenome (putatively derived from P2, the unknown progenitor). (TIF) [file pgen.1010226.s001.tif]

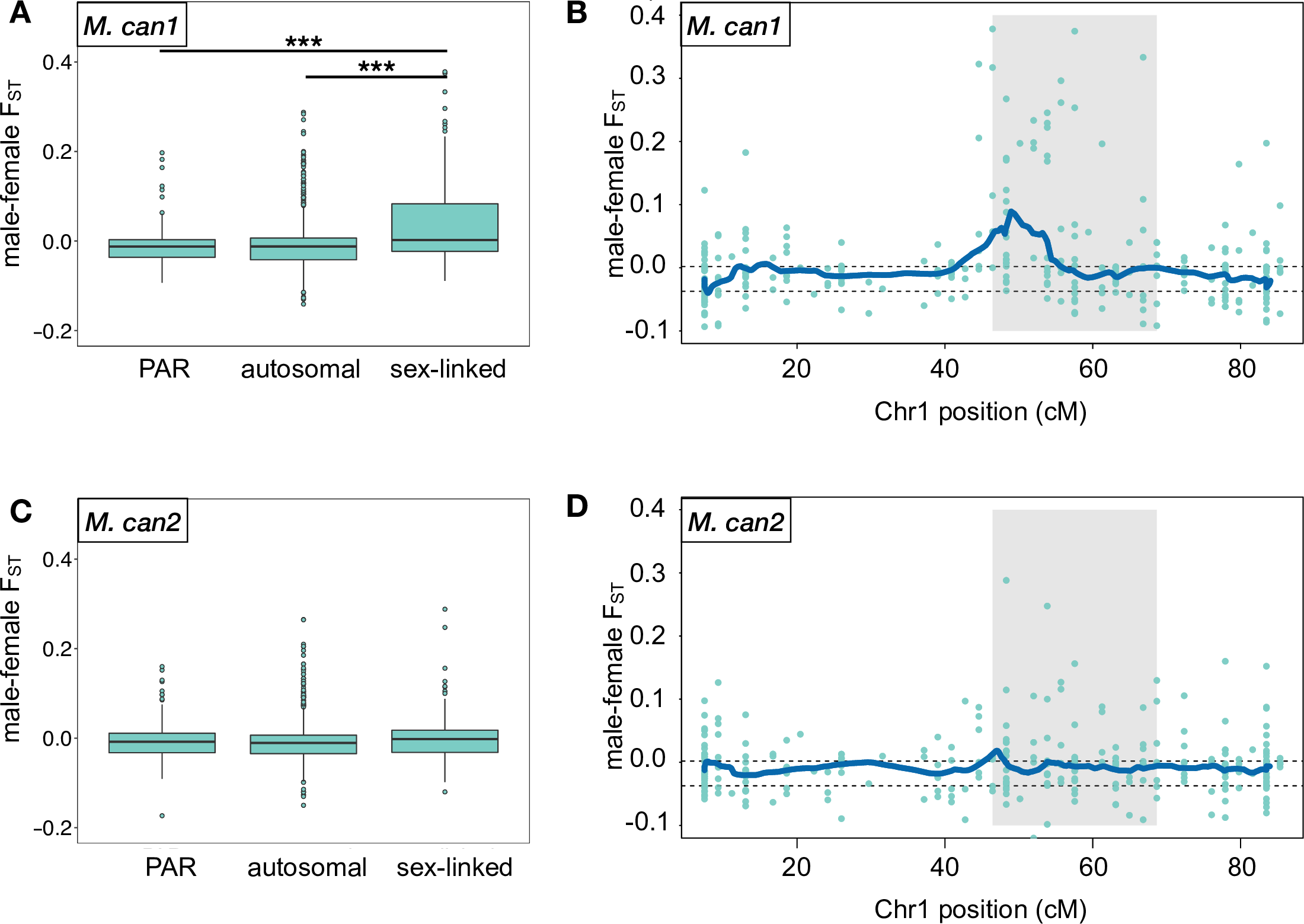

Supplement: S2 Fig — (A) Boxplot of FST between males and females in the M. can1 subgenome, with autosomal, pseudoautosomal, and sex-linked categories defined according to the linkage map for M. annua. (B) Rolling average of 20 transcripts across linkage group 1 in the M. can1 subgenome. Grey shaded regions indicate the sex-linked region inferred for M. annua. Horizontal dashed lines show 95% CI based on comparison with autosomes. (C) Boxplot of FST between males and females in autosomal, pseudoautosomal, and sex-linked regions in the M. can2 subgenome. (D) Rolling average of 20 transcripts across linkage group 1 in the M. can2 subgenome. (TIF) [file pgen.1010226.s002.tif]

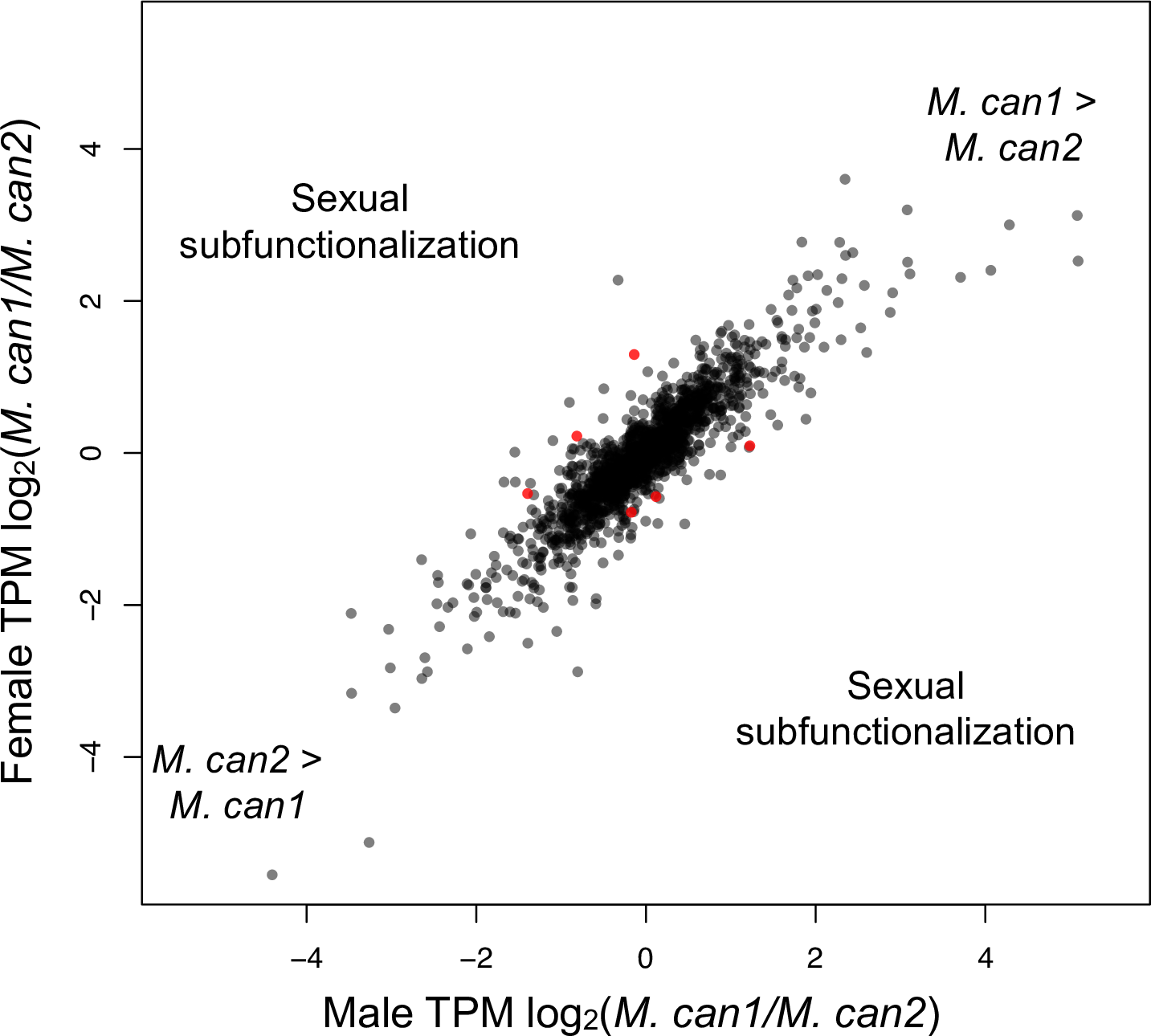

Supplement: S3 Fig — Expression is shown in TPM (transcripts per million). Several homeolog pairs are subfunctionalized (expressed higher in one subgenome). Six pairs of homeologs showed evidence of sexual subfunctionalization (shown in red). (TIF) [file pgen.1010226.s003.tif]

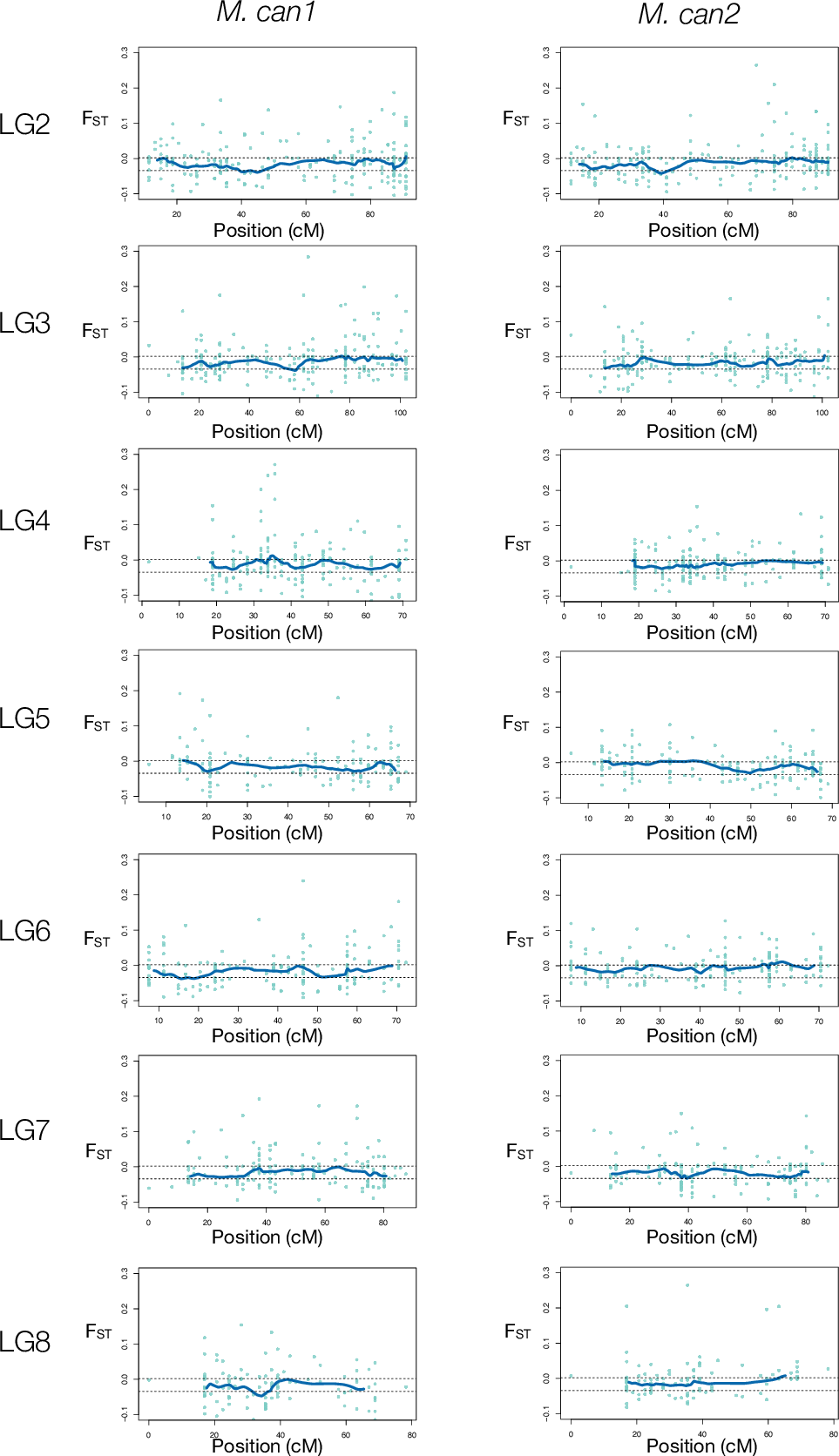

Supplement: S4 Fig — FST in rolling windows of 20 transcripts across linkage groups 2–8 in both the M. can1 and M. can2 subgenomes. Horizontal dashed lines show 95% CI. (TIF) [file pgen.1010226.s004.tif]

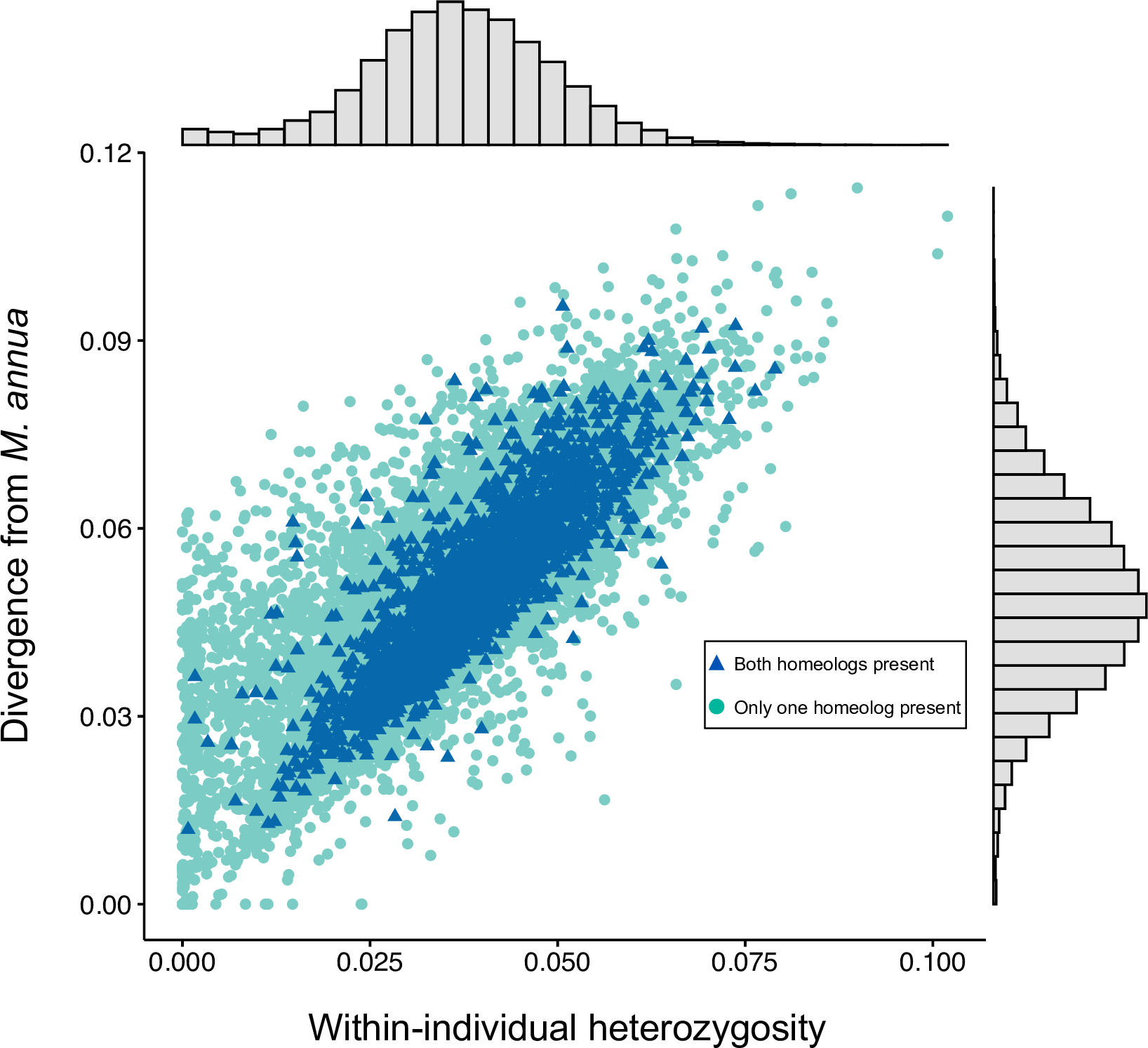

Supplement: S5 Fig — Blue triangles show values for transcripts in which both homeologs are present, whereas green circles show values when no homeolog is identified for a transcript. We hypothesized that when only the M. can1 homeolog was present, within-individual heterozygosity and divergence from M. annua should be low, while when only the M. can2 homeolog was present, within-individual heterozygosity should be low while divergence with M. annua should be high. However, because there are no two distinct distributions in the divergence from M. annua, it is not possible ¨to assign single homeologs to subgenomes. (TIF) [file pgen.1010226.s005.tif]

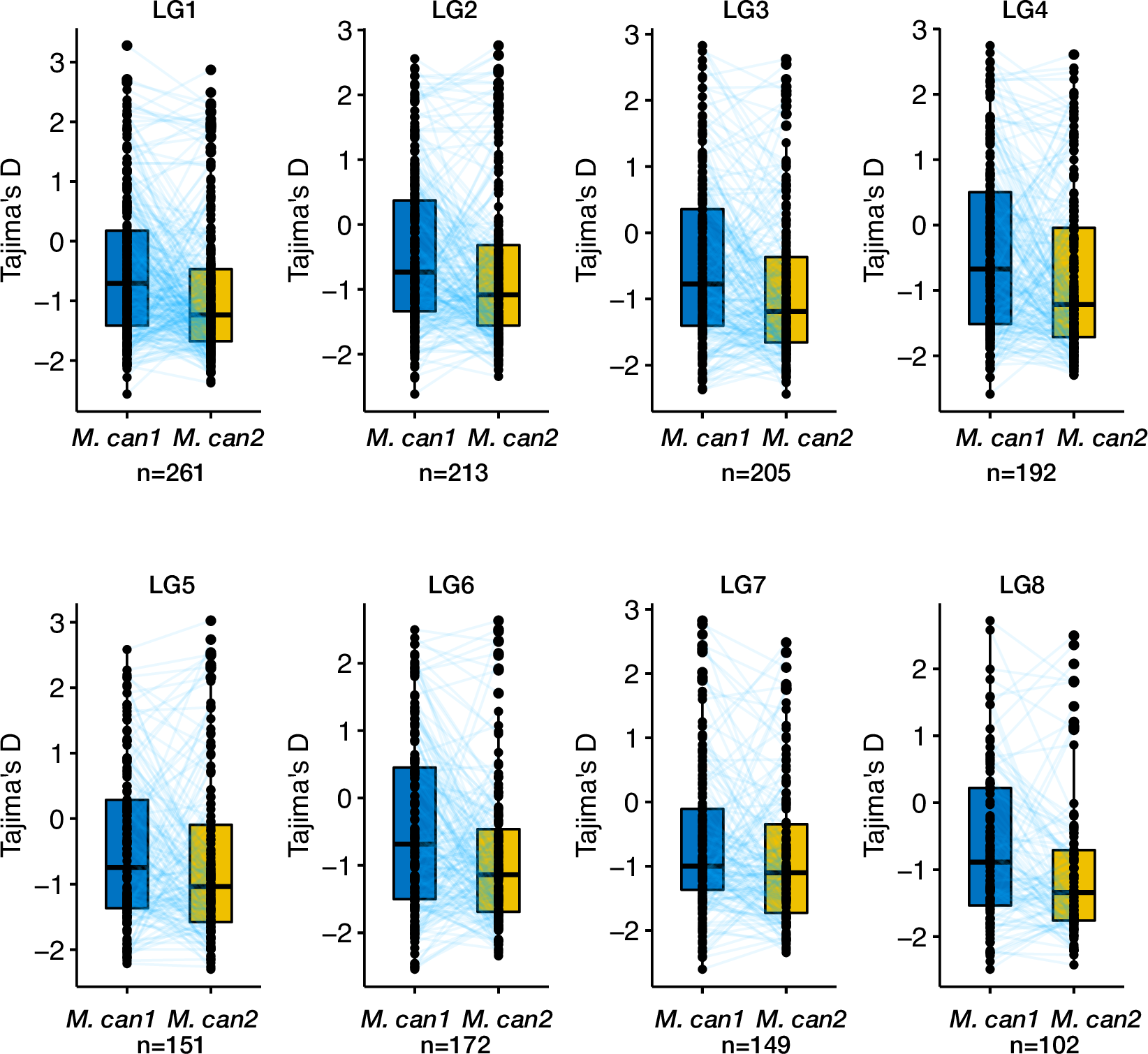

Supplement: S6 Fig — (TIF) [file pgen.1010226.s006.tif]

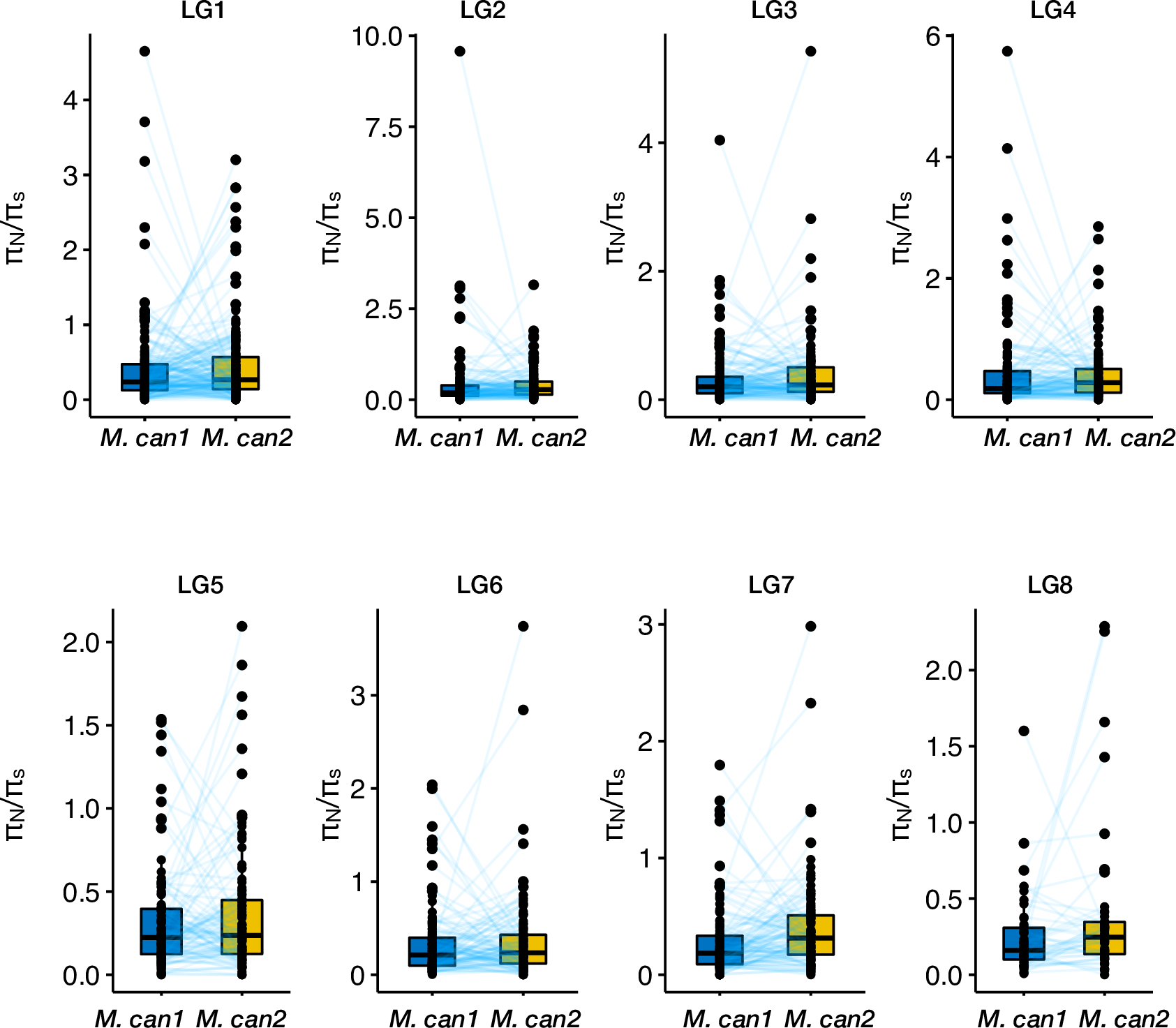

Supplement: S7 Fig — (TIF) [file pgen.1010226.s007.tif]

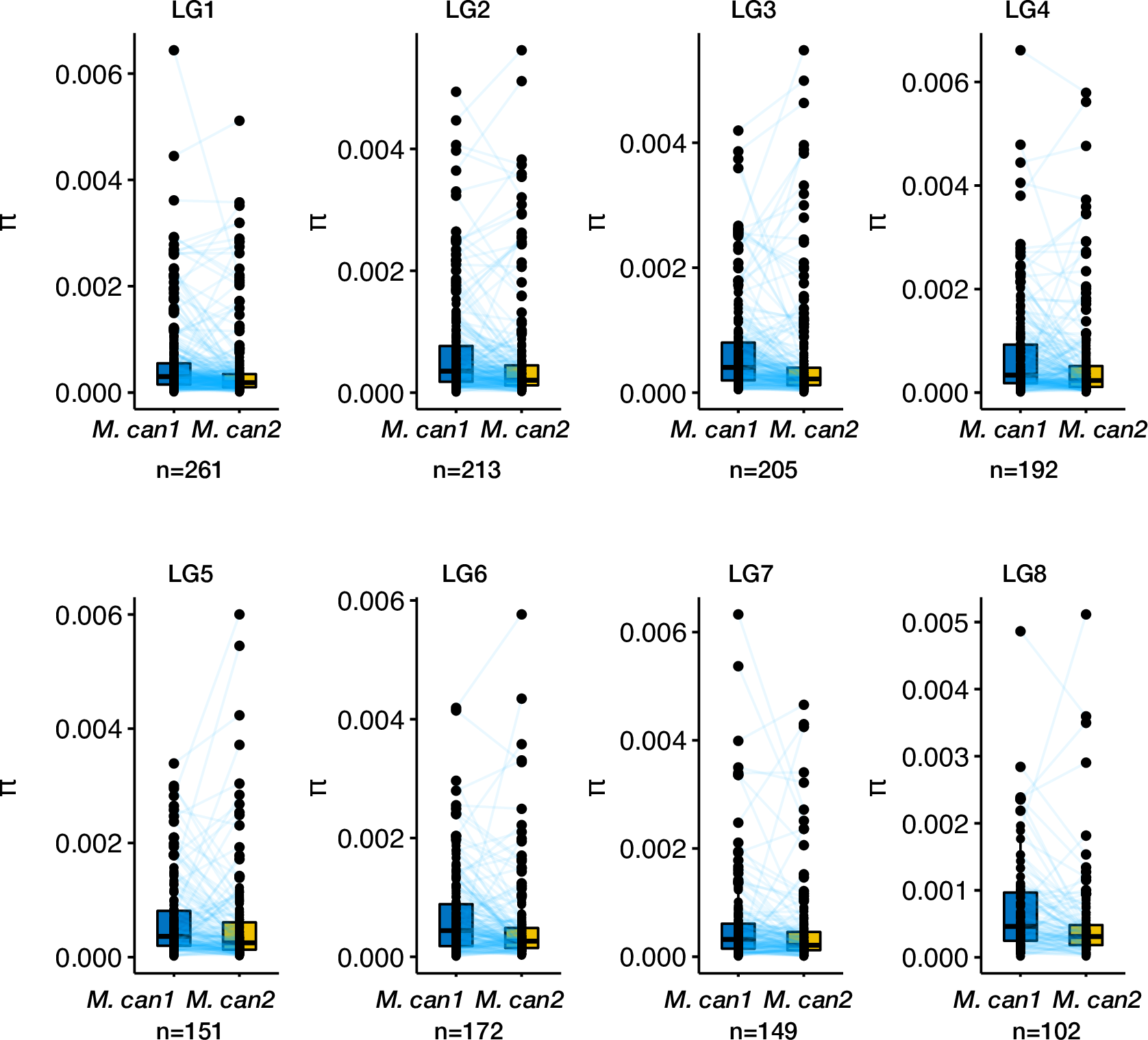

Supplement: S8 Fig — (TIF) [file pgen.1010226.s008.tif]

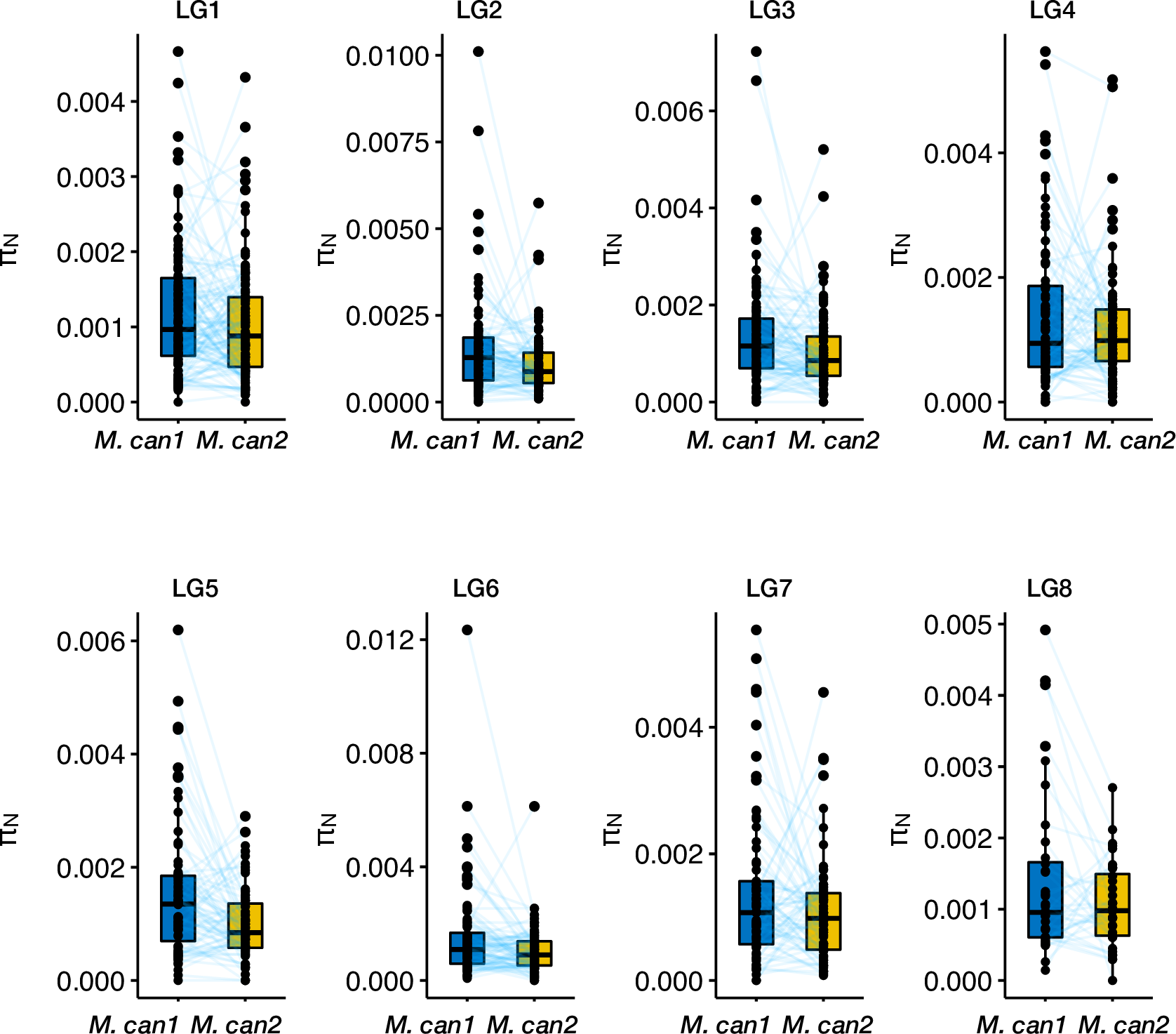

Supplement: S9 Fig — (TIF) [file pgen.1010226.s009.tif]

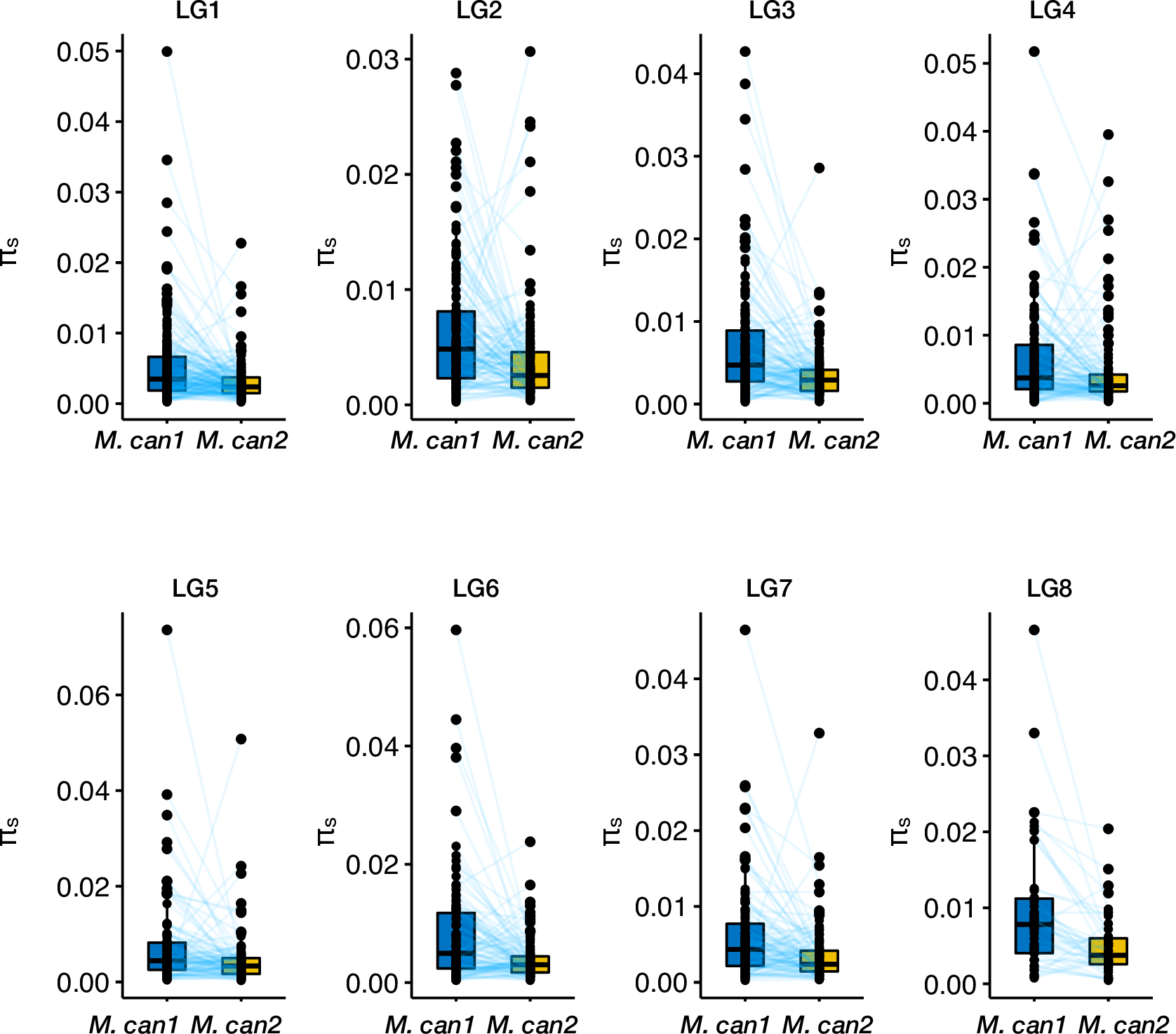

Supplement: S10 Fig — (TIF) [file pgen.1010226.s010.tif]

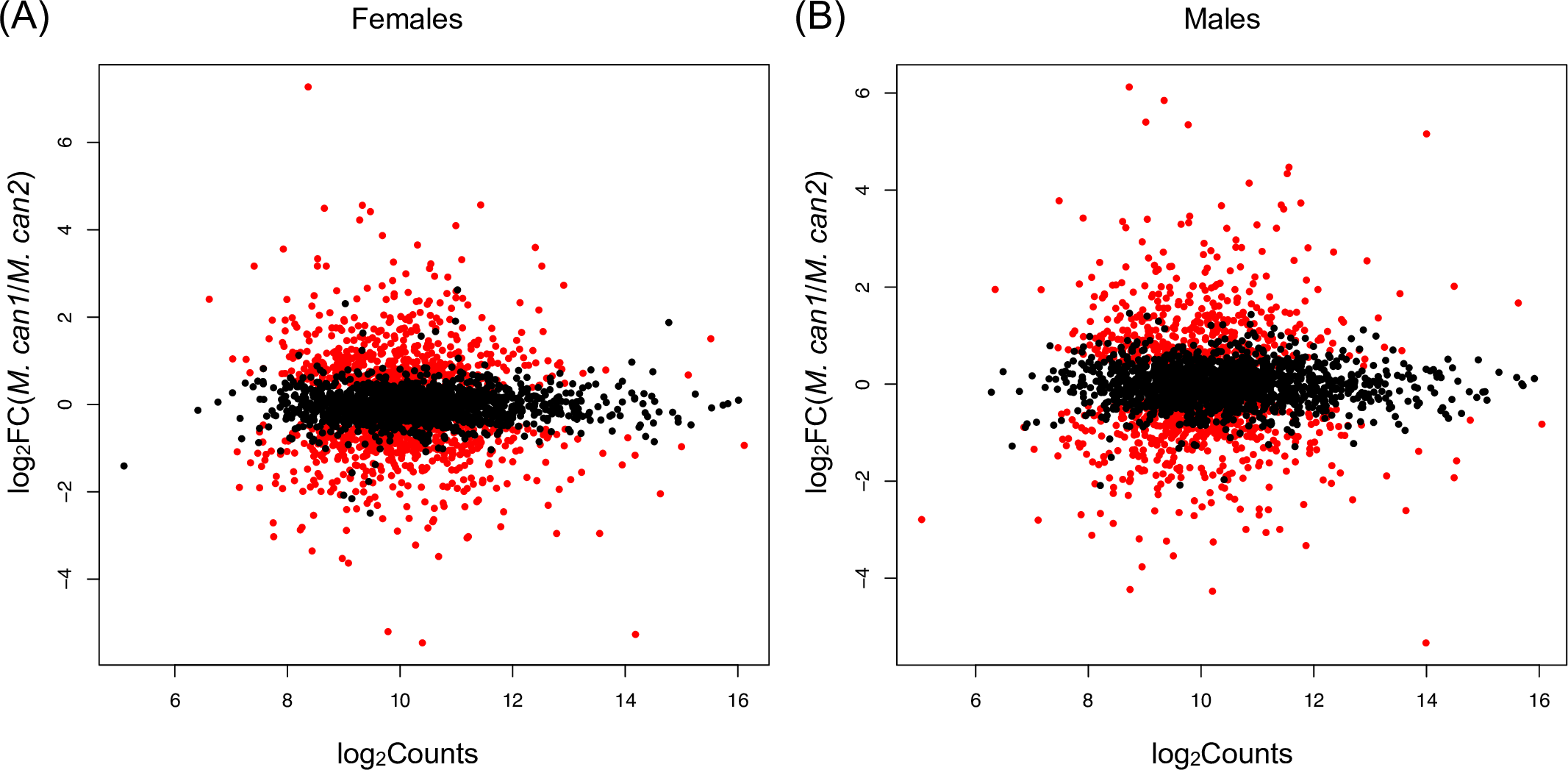

Supplement: S11 Fig — Genes that are significantly differently expressed between subgenomes are shown in red. (TIF) [file pgen.1010226.s011.tif]

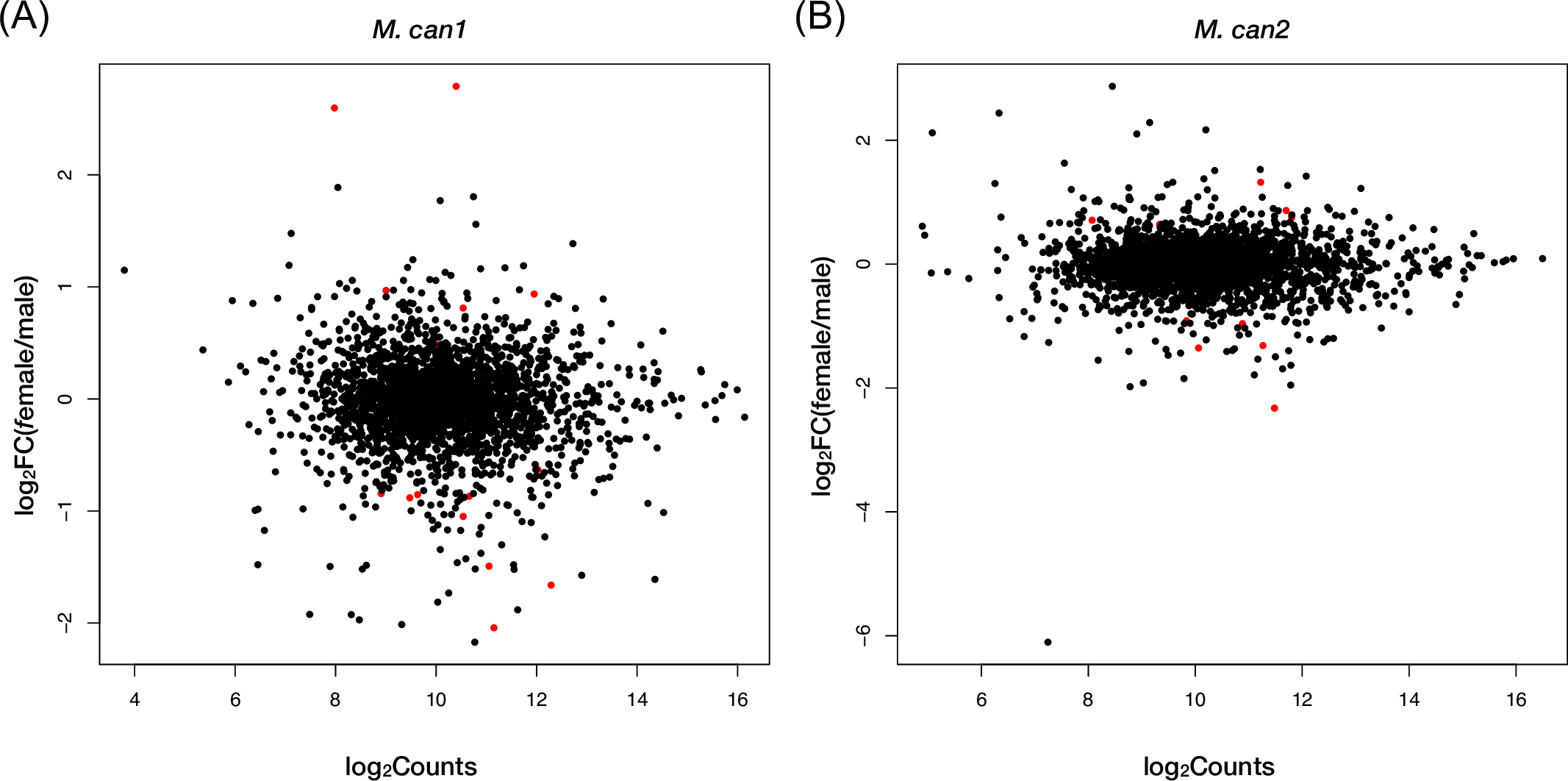

Supplement: S12 Fig — Genes that are significantly differently expressed between the sexes are shown in red. (TIF) [file pgen.1010226.s012.tif]

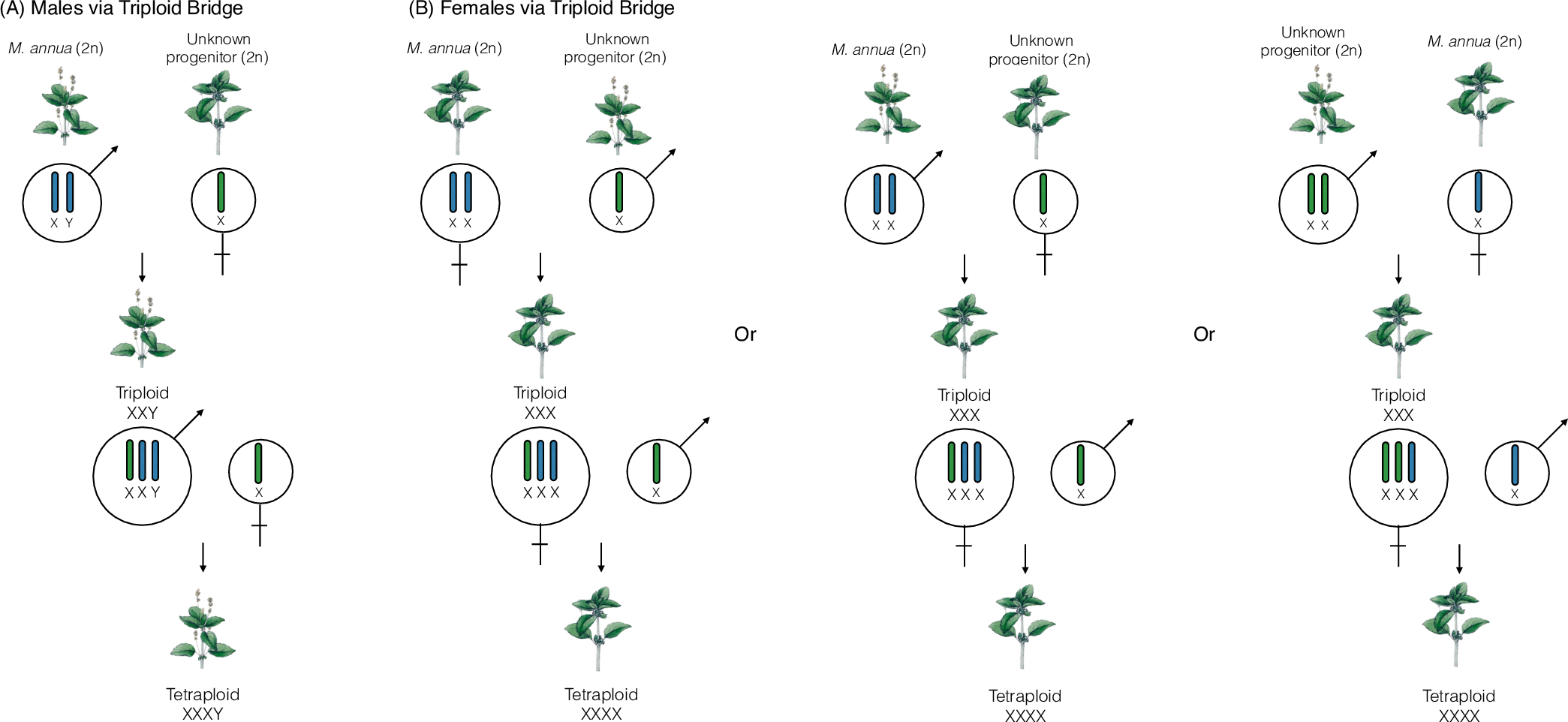

Supplement: S13 Fig — M. canariensis most likely formed via a triploid bridge, which requires at least two hybridization events: one resulting in males and one resulting in females. Males most likely evolved when an unreduced gamete from diploid, male M. canariensis fused with a haploid gamete containing the X chromosome of the unknown progenitor species. This resulted in a triploid offspring, which backcrossed with a haploid gamete containing the X chromosome from the unknown progenitor species. Females also likely formed via a triploid bridge pathway, though there are at least three possible crossing scenarios that result in the XXXX genotype. Figure adapted from [114]. (TIF) [file pgen.1010226.s013.tif]

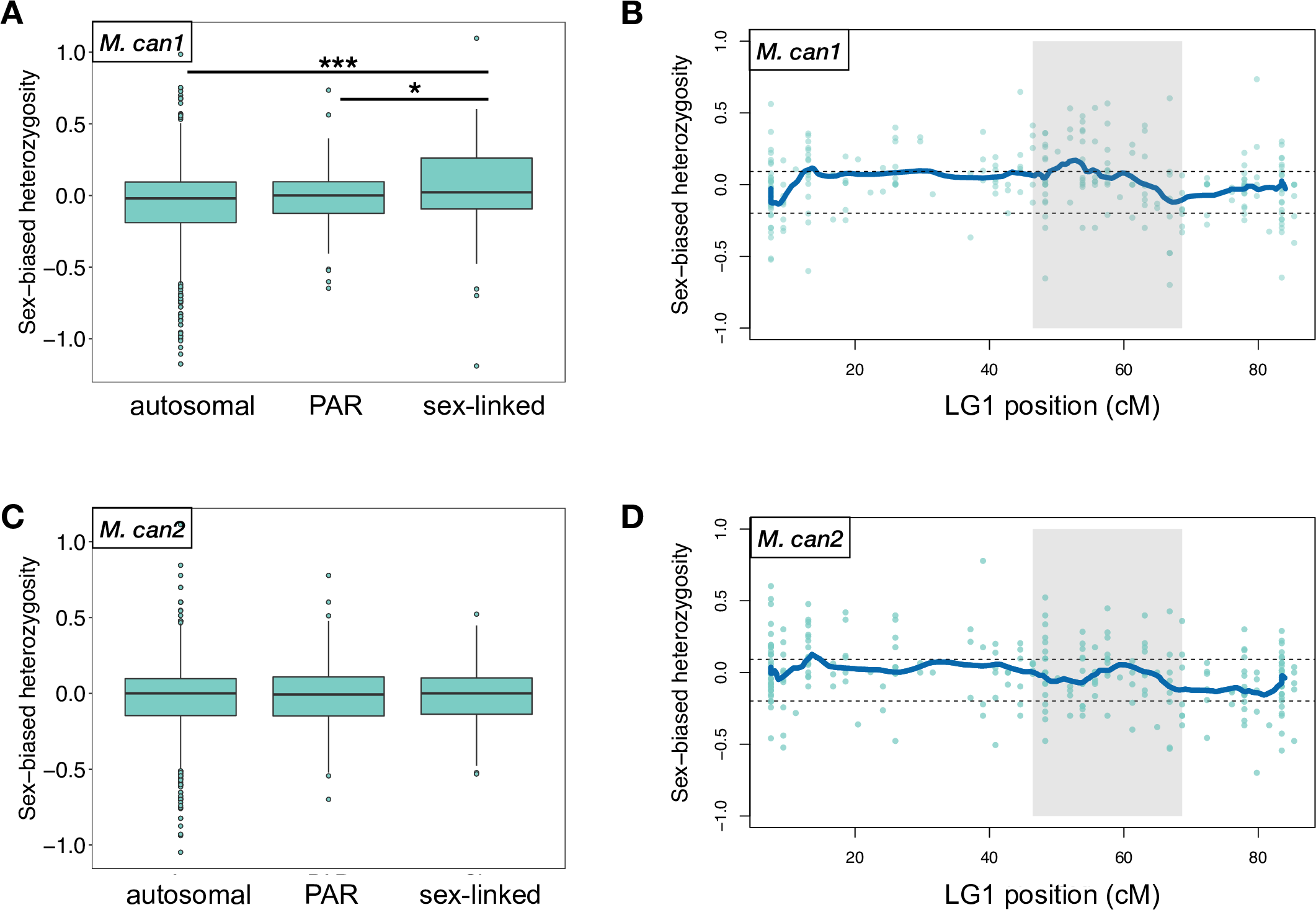

Supplement: S14 Fig — (A) Boxplot of SBH between males and females in the M. can1 subgenome, with autosomal, pseudoautosomal, and sex-linked categories defined according to the linkage map for M. annua [60]. (B) Rolling average of 20 transcripts across linkage group 1 in the M. can1 subgenome. The grey rectangle indicate the sex-linked region in M. annua [60]. Horizontal dashed lines show 95% CI based on comparison with autosomes. (C) Boxplot of SBH between males and females in autosomal, pseudoautosomal, and sex-linked regions in the M. can2 subgenome. (D) Rolling average of 20 transcripts across linkage group 1 in the M. can2 subgenome. (TIF) [file pgen.1010226.s014.tif]
